# Behavioral savings sessions increase the pursuit of solar products among refugees in Uganda

**DOI:** 10.1038/s44168-025-00212-x

**Published:** 2025-03-29

**Authors:** Sarah Elven, Jorge Luis Castañeda Núñez, Samantha de Martino, Michelle Dugas, Sayan Kundu

**Affiliations:** 1 Grantham Research Institute on Climate Change and the Environment, London School of Economics, London, UK; 2 The World Bank, Washington, DC USA

**Keywords:** Economics, Psychology, Developing world

## Abstract

De facto exclusion of vulnerable populations from markets for energy-efficient technologies can result in multiple barriers to access. For example, exclusion can lead to limited knowledge about available products, an inability to distinguish high-quality from low-quality devices, and limited options for financing, making products seem unobtainable. However, behaviorally informed interventions can offer promising solutions in such contexts, even where exclusion is the result of structural causes. This paper uses a randomized control trial to consider the potential of such interventions for refugees in Uganda in the context of certified solar markets. We evaluate a behaviorally-informed information and savings session embedded in Village Savings and Lending Association (VSLA) meetings, finding evidence for increased pursuit of certified solar products in the treatment group two months later. Results manifest through the barriers described, with increased knowledge, trust in solar companies, financial inclusion through savings group support, and aspirations mediating effects.

## Introduction

Access to clean, safe, and reliable energy sources is a crucial issue to address both poverty and climate change. Approximately 80% of those currently living without access to electricity are from Sub-Saharan Africa, with over 600 million people in the region (47% of the population) not connected to the grid^1^. Though progress is being made worldwide, it is slowest in Sub-Saharan Africa, with the region expected to represent 85% of the 660 million worldwide without electricity access in 2030. Energy access is even worse for more marginalized populations, such as refugees living in settlements, amongst whom 97% do not have electricity access^2^.

Households without electricity largely burn fossil fuels to produce energy (wood and charcoal are used for cooking amongst 91.8% of Ugandans) and on piecemeal measures for lighting, such as paraffin, candles, dry cell batteries, and torches (~40% of Ugandans use these sources, and an even greater percentage among the poor)^3^. This has environmental implications, with solid fuels used for cooking and heating contributing 25% of global atmospheric black carbon^4^ emitted globally, as well as to other greenhouse gas emissions such as carbon dioxide, methane, and nitrogen dioxide^5,6^. Fuel collection for burning in the home can also add to the deforestation and degradation of local landscapes, especially in densely populated areas^7^. In addition to these environmental impacts, reliance on non-renewable and low-quality fuel sources can place financial pressure on households^8,9^ and reduce economic opportunities^10^, educational outcomes^11^, safety^12^, and quality of life^13,14^.

In the absence of grid connectivity, off-grid solar devices offer a clean, safe, and reliable alternative energy source. However, access to these devices is limited in many parts of Sub-Saharan Africa due to various constraints. In particular, product cost^15^, market uncertainty, customer remoteness, and regional instability pose barriers to establishing reliable solar supply chains across parts of the African continent^16^. As with grid electricity, barriers to off-grid solar products are especially binding among refugee populations. This is particularly true of certified solar devices (i.e., products that meet quality guidelines set by independent associations, such as VeraSol, or industry standards, such as those defined by the International Organization for Standardization) due to the fact that refugees tend to live in settlements, away from host communities, and are rarely targeted explicitly for certified solar devices^17^. This de facto exclusion has several implications for how much refugees value solar products, their expectation that ownership of solar products is an achievable goal, and, ultimately, their efforts to adopt quality solar.

First, exclusion can result in a lack of knowledge of the possible benefits of solar devices for refugees, including opportunities to save money on energy in the long run and even generate income with more powerful solar devices. This lack of knowledge can lead to a low valuation of solar products by refugees^18^.

In addition, the relative lack of certified solar products in settlements can result in the proliferation of cheaper, less reliable devices, with little available information on how to distinguish them from high-quality certified products. This asymmetric information can diminish refugees’ perceptions of the quality of solar products and their trust in even reputable vendors^18^.

Third, refugees perceive high-quality certified solar products to be unaffordable and thus unattainable, especially given limited access to formal financing. Without national identification or easy access to labor markets, refugees often have low or unstable incomes. While some solar companies have piloted solar financing, such as pay-as-you-go schemes, in settlements (with the support of international aid), products often have since been withheld or withdrawn due to perceived default risk for this population^17^. Even where financing options are available, interest rates and loan terms can be prohibitive for poor households^19^. Groups such as Village Saving and Lending Associations (VSLAs)—common social groups among refugees in Uganda for saving and borrowing money for productive investments—could offer an alternative to formal financing of solar products. However, they do not usually lend money or encourage targeted savings for solar, partly due to the low salience of the possible productive benefits of solar devices. This lack of viable funding options, alongside low and unpredictable income streams, can lead refugees to consider high-quality solar devices to be out of their reach^18^. If they do not believe they are able to achieve solar product ownership (low self-efficacy), refugees may not allow themselves to even aspire to purchase solar devices.

While structural issues are undoubtedly a source of these barriers to solar adoption, refugees’ behavioral responses are also important to consider. Indeed, though structural solutions like pro-poor subsidies can be critical to promoting adoption of welfare-improving technologies^19,20^, evidence suggests that these alone may not yield desired impacts on investment and sustained use of technology^21^. Existing work suggests the potential for behaviorally-informed support in both the short and long term. In the short term, interventions such as the one evaluated in this paper may offer immediate improvements in contexts where structural changes are expensive and slower to materialize. Over time, as structural issues in the solar landscape improve, companies and policies that operate with an evolved understanding of human behavior may see desired gains in energy-efficient technology adoption^18,22^.

In this paper, we test a behaviorally informed intervention to alleviate barriers for refugees in Uganda—low knowledge of products, low trust in solar vendors, and few financial options for purchase—to improve access to and, ultimately, sustainable adoption of solar technologies. Uganda is in some ways a unique setting, since it hosts the largest number of refugees in Africa, in part because it has some of the most accommodating national policies for refugees^23^. However, refugees still may lack the same benefits as citizens unless they are able to obtain a national ID and move out of settlement areas, both of which are very challenging, even within Uganda^23^. The result is a constrained environment for opportunities, resulting in cycles of poverty and relative exclusion.

Our paper evaluates the impact of an intervention on the pursuit of high-quality solar products within the context described. Using a randomized controlled trial, we leverage trusted community channels in refugee settlements—312 VSLAS—to diffuse information on solar products among members and to alleviate financial barriers. Specifically, the intervention consists of a one-off session embedded in each of the selected VSLAs’ regularly scheduled meetings, delivering (1) salient information to members on the savings and productive benefits of solar devices (with the support of behaviorally informed flyers); (2) support to identify and access high-quality (certified) solar products; and (3) training and support to establish an attainable savings goal, paired with public verbal commitment to the VSLA cohort. Treatment was administered during the early months of the annual group savings cycle, and data collection occurred three months later, approximately in the middle of the cycle. The outcomes evaluated relate to the pursuit of solar products: specifically, savings for solar and contact with solar companies, as well as ownership of solar products. Mediation analysis then examines whether effects were mediated by reducing the barriers mentioned above: knowledge of high-quality solar products, trust in solar companies, and a sustainable alternative financing design.

Our main findings are as follows. First, treatment resulted in a greater pursuit of solar products. Treatment individuals were 32 percentage points more likely to have a savings goal for a solar product and 13 percentage points more likely to be saving consistently (weekly) for a solar home system (a larger, multi-use solar product) at endline. They were also saving approximately 2100 Ugandan shillings more per week toward a solar product goal (+$0.57 or +136%) than the control group at endline, an amount corresponding to around ~30% of weekly savings as measured at baseline, and to a minimum of 1% of household income for a household of four living on the poverty line of $2.15 per day (~8000 UGX)^24^. (Around 70% of refugees in 2018 lived below this line, and likely a greater proportion since COVID-19, since recovery from the pandemic has been slower for the refugee population^25^). Individuals within treated VSLAs were also 7 percentage points more likely to have contacted companies selling certified solar products since the intervention.

Given the significant investment needed by poor households for these products, we do not yet see increases in solar ownership in our sample during the short period of study. Ownership is not an outcome for which we expected to observe impacts, given our short-term focus; individuals would need to save for multiple months to accumulate the savings needed to purchase relatively costly certified solar products.

Through mediation analysis, we find evidence that increases in the pursuit of solar were partly mediated by an increase in product knowledge. This finding emphasizes the importance of overcoming information asymmetries for energy transition, a constraint that is likely magnified for marginalized populations. However, the mediation analysis also points to the importance of addressing barriers beyond information. In particular, we find the social support of participants’ VSLA group was instrumental in mediating the impacts of the intervention. The informal groups particularly made a difference at the beginning of individuals’ savings journeys for solar—perhaps helping them research solar products to establish a savings goal. Finally, we find that positive impacts on savings goals and amounts saved were in part mediated through increased aspirations for solar, suggesting that links between poverty exclusion and aspirations should not be overlooked when designing interventions to boost technology adoption among vulnerable populations.

This paper offers several contributions to the literature at the intersection of behavioral economics and climate action in the developing world. In general, it adds in numerous ways to the literature on barriers to energy-efficient technology adoption—a crucial obstacle to be addressed for technological advances to help mitigate climate change^26^.

We provide evidence of the benefits of simple, cost-effective behavioral solutions to address barriers to the pursuit of high-quality solar products by refugees. Though existing work on barriers to technology adoption points to the importance of behavioral constraints^27^, the literature on solar devices is relatively scarce. Studies on solar adoption in the developed world do find evidence of the importance of behavioral solutions, such as social learning^28^, and salience of valuable product characteristics^29^. However, these studies do not extend to the context of refugees in settlements in the developing world, who arguably face numerous additional barriers to solar access, pursuit, and adoption.

The study also contributes to the literature on the low valuation of “welfare-improving” technologies in the developing world by consumers, a phenomenon widely observed in the healthcare, agriculture, and energy-efficiency literatures^18,27,30–34^. We consider the role of market exclusion in the low valuation of solar benefits by refugees. Drawing on existing literature in this area^34–37^, we ask whether making salient the benefits of high-quality, certified solar products to refugee consumers can increase the perceived relevance of solar products, thereby increasing demand. Though similar approaches have yielded positive effects in different contexts^29^, this paper offers the first empirical test of such an intervention among a marginalized population in a developing country.

Also related to the literature on the low valuation of new technologies, the paper contributes to work on markets for “lemons” in the developing world^38^. These can occur in the presence of asymmetric information about product quality, leading to a situation in which low- and high-quality goods cannot be differentiated, and to a low willingness to pay for both goods. Literature on this issue in developing countries is scarce, with existing studies on solar markets reaching mixed conclusions. Indeed, while early work finds no evidence for greater cost-effectiveness of quality branded products for poor consumers^39^, recent studies identify a “two-tiered” solar market (in Malawi^40^) and conclude that concerns about product quality are an important barrier to solar adoption (in Senegal^41^). Work to alleviate the barriers caused by a market for lemons in the context of solar finds that durability signals, such as certification and warranties, can increase willingness to pay among more experienced buyers^41^. However, the literature also stresses that purveyors of certified products do not yet go far enough to inform consumers about the possible impacts of their choices, to offer sustainable financing options, or to prevent the waste that occurs after warranty periods are over^40,42^. Our work contributes to this literature in a context where information asymmetries are exacerbated. Further, we broaden the scope of existing empirical studies beyond the more common solar lanterns to include the adoption of larger, more powerful solar products (e.g., solar home systems). As such, we also test the importance of addressing additional barriers, such as financial exclusion and savings constraints.

Finally, this study contributes to the considerable literature on behavioral savings barriers for those excluded from formal financial markets, and in particular the value of goal-setting and soft-commitment interventions in the context of informal savings groups. Contributions to this literature are important for behavioral economics and climate action for two reasons. First, due to challenges with loans and pay-as-you-go schemes in new markets for energy-efficient devices^39,40,42^ and financial exclusion in much of the developing world, the diffusion of these products in the short- and medium-term may partly rely upon individuals’ ability to save for them. Second, and more broadly, behavioral savings barriers speak, in part, to the difficulty of habit formation in the context of scarcity (poverty) and may therefore have broader implications for sustained climate-relevant behavioral change.

Our contribution to the behavioral savings literature combines insights on the challenges that commitment^43,44^ and aspirations can pose for consistent savings in a context of scarcity. When regular saving is challenging, mechanisms requiring some form of commitment can have positive impacts^45–47^, though savings flexibility can still be important for households with unpredictable incomes and expenses. Moreover, informal group saving for a specified goal can yield larger amounts than putting money aside individually^47^, and public commitment to a goal within group settings can be a powerful “soft” commitment vehicle due to peer observability^48^.

Where scarcity leads to aspiration traps, resulting in more modest goals and lower savings effort^49–51^, psychological interventions, such as boosting aspirations or the self-efficacy of excluded communities^52^, as well as participation in thoughtful goal-setting exercises^53^, can be beneficial. To avoid overly high aspirations, which can lead to frustration, discouragement, and ultimately abandonment of aspiration, it is necessary to promote realistic goals that can be achieved with effort^54–56^.

Our contribution combines these insights to consider the value of realistic goal-setting and soft commitment within informal savings groups in the context of low solar saving caused by low aspirations and self-efficacy in a marginalized population. By focusing on group savings mechanisms rather than loans or pay-as-you-go systems, we empirically test a strategy to de-risk solar financing for vulnerable populations.

## Results

### Results: outcomes

The OLS results presented below are robust to models specific for binary dependent variables, an ANCOVA specification, a difference-in-differences specification, and corrections for multiple hypothesis testing. (See Supplementary Materials for robustness tests and adjusted *p*-values.)

Tables 1 and 2 display the results of our OLS estimates for the impact of being a member of a VSLA assigned to the treatment group. Table 1 presents impacts on participants’ pursuit of solar products, and Table 2 presents estimates for impacts on participants’ ownership of these products. Outcomes in both tables are dichotomous variables, and results are robust to specifications for binary dependent variables (Logit: Supplementary Tables 5 and 6).

**Table 1:** OLS regressions for intervention impact on pursuit of solar products

	(1)	(2)	(3)	(4)	(5)
	Savings goal	Solar savings goal	Track savings goal	Contacted company	Expects to purchase this year
Treatment VSLA	0.02	0.32***	0.02	0.07***	0.07
	(0.02)	(0.04)	(0.05)	(0.02)	(0.04)
Replacement	0.01	−0.13***	−0.02	−0.03	−0.00
	(0.03)	(0.04)	(0.05)	(0.02)	(0.05)
Constant	0.83***	0.23***	0.52***	0.11**	0.57***
	(0.04)	(0.07)	(0.09)	(0.04)	(0.08)
VSLA controls	Yes	Yes	Yes	Yes	Yes
Individual control	Yes	Yes	Yes	Yes	Yes
SEs clustered VSLA	Yes	Yes	Yes	Yes	Yes
R-squared	0.03	0.21	0.02	0.03	0.01
Observations	1186	1186	1186	1186	1186

Each column presents the results of OLS regressions for binary dependent variables on the binary treatment assignment variable. Results are ITT estimates due to imperfect intervention compliance. All regressions control for VSLA-level controls (a settlement dummy, a dummy for VSLAs that meet at least weekly, the number of members in the VSLA, and the VSLA’s share price (normalized to weekly)), and individual-level controls (dummy for at least primary education). Robust standard errors clustered at the VSLA level are displayed below coefficients in parentheses. Asterisks denote a statistically significant difference at the 1% ***, 5% **, or 10% * levels.

**Table 2:** OLS regressions for intervention impact on the ownership of solar devices

	(1)	(2)	(3)	(4)	(5)	(6)	(7)
	No solar device	Panel or battery components	Lantern: no charging	Lantern: with charging	Solar home system	Larger solar device	Acquired solar since intervention
Treatment VSLA	0.03	0.01	−0.01	−0.02	−0.01	−0.03	−0.03
	(0.04)	(0.03)	(0.03)	(0.01)	(0.02)	(0.03)	(0.02)
Replacement	−0.01	0.02	−0.02	0.02	−0.01	−0.00	−0.05
	(0.04)	(0.03)	(0.03)	(0.02)	(0.02)	(0.04)	(0.03)
Constant	0.64***	0.04	0.18***	0.08***	0.08***	0.26***	0.11**
	(0.07)	(0.04)	(0.05)	(0.03)	(0.02)	(0.05)	(0.05)
VSLA controls	Yes	Yes	Yes	Yes	Yes	Yes	Yes
Individual control	Yes	Yes	Yes	Yes	Yes	Yes	Yes
SEs clustered VSLA	Yes	Yes	Yes	Yes	Yes	Yes	Yes
R-squared	0.03	0.01	0.01	0.02	0.01	0.02	0.04
Observations	1186	1186	1186	1186	1186	1186	1186

Each column presents the results of OLS regressions for binary dependent variables on the binary treatment assignment variable. Results are ITT estimates due to imperfect intervention compliance. All regressions control for VSLA-level controls (a settlement dummy, a dummy for VSLAs that meet at least weekly, the number of members in the VSLA, and the VSLA’s share price (normalized to weekly)), and individual-level controls (dummy for at least primary education). Robust standard errors clustered at the VSLA level are displayed below coefficients in parentheses. Asterisks denote a statistically significant difference at the 1% ***, 5% **, or 10% * levels.

From Table 1, we see that, compared to individuals in the control VSLA groups, those in treatment VSLAs were, on average, more likely to have a solar product savings goal (32 percentage points) and to have contacted a certified solar product company during the study period (7 percentage points). They were not more likely to have a savings goal in general, to track their goals, or to expect to purchase solar in the coming year, however.

Table 2 presents estimates for impacts on participants’ ownership of solar products. Note that though the outcome of interest is certified solar, we do not specify this directly in the survey, but rather ask generally about solar ownership. If we had observed any differences here, we could have then dug into the specifics of the make and model of any solar devices acquired to ensure these devices were certified. However, we cannot reject the hypothesis of no significant differences between treatment and control for outcomes on solar product ownership.

The regressions on outcomes relating to the pursuit of solar in Table 3 use a Tobit specification due to the censored nature of these outcome variables. Outcomes in regressions (1)-(4) refer to the specific products individuals are saving toward and are measured as dichotomous variables. Those in regressions (5)–(7) relate to weekly savings toward solar in shillings and are continuous. When it came to solar savings, individuals from treatment VSLAs were saving approximately 2120 shillings per week (USD 0.57) more toward their solar goal, specifically for products more commonly sold by certified companies (lanterns and solar home systems).

**Table 3:** Tobit regressions for intervention impact on the pursuit of specific solar products

	(1)	(2)	(3)	(4)	(5)	(6)	(7)
	Panel battery components	Lantern: no charging	Lantern: with charging	Solar home system	Weekly solar savings: VSLA	Weekly solar savings: Other	Weekly solar savings: Total
Treatment VSLA	0.03*	0.10***	0.09***	0.13***	1888***	1104***	2120***
	(0.02)	(0.02)	(0.03)	(0.03)	(262.08)	(328.89)	(295.93)
Replacement	−0.02	−0.01	−0.09**	−0.06*	−740.6**	−700.9**	−942.1**
	(0.02)	(0.03)	(0.04)	(0.03)	(326.14)	(347.97)	(384.02)
VSLA controls	Yes	Yes	Yes	Yes	Yes	Yes	Yes
Individual control	Yes	Yes	Yes	Yes	Yes	Yes	Yes
SEs clustered VSLA	Yes	Yes	Yes	Yes	Yes	Yes	Yes
Observations	1186	1186	1186	1186	1161	749	1184

Each column presents the results of Tobit regressions for dependent variables on the binary treatment assignment variable. Dependent variables are binary in columns 1–4 and continuous in 5–6. Results are ITT estimates due to imperfect intervention compliance. All regressions control for VSLA-level controls (a settlement dummy, a dummy for VSLAs that meet at least weekly, the number of members in the VSLA, and the VSLA’s share price (normalized to weekly)), and individual-level controls (dummy for at least primary education). Robust standard errors clustered at the VSLA level are displayed below coefficients in parentheses. Asterisks denote a statistically significant difference at the 1% ***, 5% **, or 10% * levels.

### Results: mediators

We next present the results of a mediation analysis, in which we consider whether any of the impacts reported were mediated through the intermediate outcomes outlined in the Theory of Change (knowledge of solar benefits, trust in solar companies, VSLA support, aspirations to own solar products, and self-efficacy for reaching solar goals). The effects of the intervention on these intermediate outcomes are shown first (Table 4), followed by the mediation analysis itself (Table 5).

**Table 4:** Intermediate outcomes

	(1)	(2)	(3)	(4)	(5)
	Knowledge: % correct	Trust in solar providers: index	VSLA Support: index	Aspire to purchase solar this year	Self-efficacy: index
Treatment VSLA	0.27***	0.20***	1.03***	0.08**	−0.08
	(0.02)	(0.06)	(0.09)	(0.04)	(0.06)
Replacement	−0.08***	0.02	−0.16	0.01	0.07
	(0.02)	(0.07)	(0.11)	(0.04)	(0.08)
Constant	0.15***	3.79***	2.74***	0.76***	2.51***
	(0.04)	(0.12)	(0.19)	(0.07)	(0.12)
VSLA controls	Yes	Yes	Yes	Yes	Yes
Individual control	Yes	Yes	Yes	Yes	Yes
SEs clustered VSLA	Yes	Yes	Yes	Yes	Yes
R-squared	0.27	0.04	0.25	0.03	0.07
Observations	1186	1137	1164	1186	1184

The table presents the results of OLS regressions to estimate the impact of treatment assignment on the five intermediate outcome variables used in the mediation analysis. All the intermediate outcome variables are indices (see Table 1) except for the aspirations variable, which is dichotomous. Results are ITT estimates due to imperfect intervention compliance. All regressions control for VSLA-level controls (a settlement dummy, a dummy for VSLAs that meet at least weekly, the number of members in the VSLA, and the VSLA’s share price (normalized to weekly)) and individual-level controls (dummy for at least primary education). Robust standard errors clustered at the VSLA level are displayed below coefficients in parentheses. Asterisks denote a statistically significant difference at the 1% ***, 5% **, or 10% * levels.

**Table 5:** Mediation analysis

Mediators	Total effect	Mediator on outcome	Mediated effect	Confidence interval		Proportion mediated
Contact solar company						
Knowledge		0.10**	0.02**	0.003	0.030	31%
Trust		−0.00	0.00	−0.005	0.006	1%
VSLA support		0.03***	0.03***	0.016	0.048	58%
Aspiration		0.03	0.00	0.000	0.004	3%
Self-efficacy		−0.02	0.00	−0.001	0.008	
Direct effect	0					
Total	0.05		0.05***	0.032	0.077	100%
**Solar savings goal**						
Knowledge		0.18***	0.10***	0.070	0.131	30%
Trust		0.10***	0.02***	0.008	0.026	5%
VSLA support		0.04**	0.03**	0.001	0.066	10%
Aspiration		0.25***	0.01**	0.000	0.023	3%
Self-efficacy		−0.04*	0.00	−0.004	0.006	
Direct effect	0.17					
Total	0.34		0.16***	0.119	0.207	49%
**Solar savings with VSLA**						
Knowledge		1826.4***	~692.2			
Trust		525.3**	~106.9			
VSLA support		69.7**	~281.9			
Aspiration		2305.1***	~176.8			
Self-efficacy		−582.4***				
Direct effect	1240					
Total	1888					

The table presents the results of the mediation analysis for the three key significant outcomes of interest (contacting a solar company, having a solar goal, and solar savings with the VSLA). OLS regressions, Tobit regressions, and seemingly unrelated regression analyses (in panels 1 and 2) are used to generate outcomes. Column 2 presents the total effect of treatment on the outcome of interest: by combining the impact of treatment on outcome, controlling for mediators (direct effect), plus the impact of treatment on outcome through the mediators (indirect effect). Column 3 presents the impacts of the mediators on the outcomes in a regression, including the treatment assignment dummy. Column 4 presents the mediated effect by combining the impact of the treatment on the mediator and the impact of the mediator on the outcome. Asterisks denote a statistically significant mediation effect at the 1% ***, 5% **, or 10% * levels, calculated using bootstrapped standard errors. Column 5 displays bias-corrected and accelerated (BCa) confidence intervals (95%) for the mediated effect. Column 6 presents the proportion mediated by dividing the mediated effect by the total effect.

Table 4 shows that the intervention had a positive and significant impact on all but one of the intermediate outcome variables. It increased knowledge of the benefits of solar products, trust in solar companies, perceptions of VSLA support, and aspirations to purchase solar. However, we cannot reject the null of no effect on savings self-efficacy in treatment VSLAs.

Estimates for the mediation of the key outcomes of interest through the intermediate outcomes are displayed in Table 5. The total effect column reports the sum of the direct effect (the impact of treatment on outcome controlling for mediators) and the indirect or mediated effect (the impact of treatment on the outcome through mediators). The mediator on-outcome column presents the coefficients on the mediators from regressing the outcome of interest on the five mediators and the treatment variable. The mediated effect column reports the indirect or mediated effect of treatment on the outcome (through the mediators).

In general, we find evidence to suggest that impacts manifest at least partially through mediators. In particular, it seems that all three effects are mediated through the solar knowledge variable, with this accounting for 31% and 30% of the impact of the treatment on contacting solar companies and having a savings goal for solar, respectively. Though the analysis for the savings variable is merely exploratory, links for each part of the causal chain appear to be strong when the knowledge variable is tested as an intermediate variable. These findings suggest that informational asymmetries were a binding constraint for the refugees in their pursuit of solar and that relevant information helped to lift this barrier to increase this.

### Contacting solar companies

In addition to mediation through knowledge about solar, impacts on contacting a solar company to find out more about their products are mediated through the VSLA social support and accountability index. The combination of the knowledge index and the VSLA support index fully mediate impacts for this outcome (i.e., all of the total effect is felt through these variables). See Supplementary Fig. 5 for a summary of the mediation model.

### Solar savings goals

In addition to knowledge, solar savings goals are mediated through trust, aspirations, and the perception of social support. These multiple mediators account for 49% of the total effect of having a solar goal. Knowledge and VSLA support account for the largest proportion of treatment on having a solar savings goal, as is the case for contacting solar companies (30% and 10% of the outcome is mediated through these variables, respectively). See Supplementary Fig. 6 for a summary of the mediation model.

### Weekly solar savings

Finally, our exploratory analysis suggests that increases in knowledge, trust, VSLA support, and aspirations positively affected weekly solar savings with participants’ VSLAs. Since the total effect (in this case, taken from our main Tobit regression in Table 3) is larger than the direct effect for this outcome, there appears to be significant mediation here, too. This is likely split across numerous intermediate variables, but determining the exact split is beyond the limits of the analysis. See Supplementary Fig. 7 for a summary of the mediation model.

## Discussion

This study sought to reduce information, trust, and affordability barriers to certified solar products in Ugandan refugee settlements. Using a solar product information and savings session delivered to informal savings groups, the intervention provided information about benefitting from solar products and identifying certified products locally and supported participants in establishing a savings goal toward an expense of their choice (solar product or otherwise).

The session positively affected participants’ pursuit of solar products. Those assigned to the treatment groups were more likely to be working toward a solar savings goal at endline and to have been in touch with companies selling certified products. Importantly, those in the treatment groups also contributed more each week to save for solar devices.

We did not find effects on solar ownership in the short term, perhaps due to the short timeframe of the study and the relatively long period needed to save for certified products (our intervention was shorter than the average time participants needed to save for the least expensive device we introduced). Since our focus in this paper was short-term habit formation and its mechanisms, follow-up data collection would be needed to verify that consistent savings led to the purchase of solar products.

Mediation analysis is used to examine the relevance of the intermediate outcomes in our Theory of Change and to explore possible mechanisms for the observed impacts. Results point to the importance of knowledge, trust, motivation, and aspirations for our observed effects. For example, impacts on the three outcomes tested are mediated through increased knowledge about certified solar products. This result emphasizes the importance of overcoming information asymmetries for clean energy transition, a constraint likely magnified for marginalized populations. In our context, VSLA groups provided a convenient platform for this diffusion of information, allowing participants to actively engage with new information together.

Beyond information, social support for participants’ savings goals through their VSLA cohort was instrumental in achieving intervention impacts. In particular, at the start of individuals’ savings journeys for solar, groups helped them research solar products and establish savings goals. The importance of VSLA social support for a solar savings goal may have been reinforced by the teamwork game played during the intervention and by the group’s public commitment to peers. Moreover, this support may have acted as a mediator for contacting solar companies by helping members reach out to providers as a group or share information after doing so separately. Though we cannot identify the precise channels, mediation through VSLA social support suggests that these groups became places where solar products are discussed and pursued – a potentially important step for de-risking financing options for these products in refugee settlements.

Though the mediation impact of aspirations is smaller than the effects of knowledge and VSLA social support, its relevance for establishing a solar savings goal and for the amount individuals save toward solar is an interesting finding. Impacts through increased aspirations for solar suggest that, especially for marginalized populations, a lack of peer role models who own solar products and a de facto exclusion from markets may have led to lower aspirations for energy-efficient technologies. Given that this sample likely had higher aspirations to purchase solar at baseline than the general population of refugees (one of the selection criteria for participants was an interest in saving toward a larger solar product), it is possible that the importance of aspirations as a mediator would be even larger in another sample. Links between poverty, exclusion, and aspirations should therefore not be overlooked when it comes to designing interventions to boost technology adoption among vulnerable populations.

The study has several limitations worth discussing. First, its focus on short-term outcomes, though valuable for modeling immediate behavioral change, precludes collecting measures on solar adoption in the long term. Data on outcomes in the longer run would be needed to ascertain whether impacts persisted and if savings for solar products translated to the purchase of solar products.

Given this limitation, we consulted existing literature on the links between intentions, initial behaviors (such as saving), and the ultimate behavior of interest (technology adoption and use) (see Supplementary Discussion 1). In summary, it is hard to glean the nature of these links in this case, since the empirical literature is limited, and eventual purchases in our sample will depend both on internal and external factors specific to the context in which we are working. Though we are hopeful that the characteristics of the savings goals made will aid goal achievement (realistic, specific, publicly committed to), it is possible that—as we observe for some respondents between intervention and endline—some refugees may need goal flexibility over time or, in more extreme circumstances, will have to set their goals aside. Note that where this is the case, we consider the VSLAs to be the most flexible, low-risk setting within which refugees can explore the feasibility of saving for solar.

Second, the fact that outcomes are self-reported could lead to measurement error or social desirability bias in responses. However, due to relatively frequent reports of setting non-solar goals and adjusting or setting aside solar goals, as well as consistent expectations about goal timelines for solar products of interest, we consider responses to be largely reliable (see Supplementary Discussion 2 for details). We also consider it unlikely that savers will misremember the amount they set aside (usually weekly) with their VSLA and thus assume measurement errors to be minimal and random.

The mediation analysis does not allow for causal interpretation, since there is no exogenous source of variation in the intermediate outcomes. Further, intermediate outcomes and dependent variables were measured at the same time, so it is not possible to verify the order of impacts as presented in the Theory of Change. Though mediation analysis is therefore exploratory, it is based on the literature and the resulting Theory of Change presented in this paper. Thus, it can still offer some insight into links between our intermediate and final outcomes, in addition to offering a helpful framework for future work that considers these barriers.

Finally, when it comes to savings interventions among acutely resource-poor populations, it is important to consider the opportunity cost of a new savings goal. Supplementary Discussion 3 asks to what extent increased solar saving occurred as a complement to or a substitute for other forms of saving in our sample, finding some evidence for substitution, though we do not have sufficient data to determine whether this reflects an optimal shift or one that could present challenges for savers down the line. Future work testing savings interventions that cover numerous possible goals or offer refugees financial support to aid their savings could further guard against any negative consequences of such trade-offs.

This study demonstrates the potential of behavioral interventions in the context of barriers to the adoption of certified solar products for refugees. The intervention had an immediate impact in a context where refugees face de facto exclusion from markets for certified solar and where structural solutions may therefore be slower to materialize. Indeed, by harnessing VSLAs as a vehicle for the pursuit of solar in a community, we find, that refugees can be induced to strive for these products even in the absence of financial incentives or changes in the local market structure. Groups allow members a space to actively engage with new information together, in addition to gaining social support and accountability within a flexible savings environment (i.e., as compared to PAYGO systems) to pursue their goals. Given these attributes of savings groups, goals with VSLAs may be more sustainable than saving or borrowing elsewhere. Further work should explore the potential impact of leveraging these informal groups when designing interventions for refugees and other excluded vulnerable populations.

## Methods

### Context: Kiryandongo and Nakivale settlements

In Uganda, electricity connectivity remains below average for Sub-Saharan Africa, with 58% of the population still off the grid in 2020^57^. Energy poverty is particularly high among the country’s nearly 1.5 million refugees, despite the country’s relatively inclusive refugee policies^8^. Indeed, as of 2018, over 75% of the Ugandan refugee population did not have access to any form of renewable energy, and many relied on firewood and kerosene. Energy represents about 22% of total household expenditures for this population, with the time burden of fuel collection at around 12–24 h per week^8^. When refugees own solar products, they tend to be small lanterns handed out by humanitarian organizations or uncertified (and often low-quality) solar products purchased in the local marketplace^58^. Thus, this study focuses on the pursuit and adoption of high-quality, certified products.

Refugee settlements in Uganda are mainly located in the West Nile region in the northwest, and in the southwest of the country. Settlements in these two areas tend to host refugees from neighboring countries, with the northwestern settlements hosting more refugees from South Sudan and the southwestern settlements hosting more individuals from countries to the south, such as the Democratic Republic of Congo (DRC), Rwanda, and Burundi. This study bases itself on one settlement from each of these areas: the Nakivale settlement in the district of Isingiro in the southwest, and the Kiryandongo settlement in the Kiryandongo district, further northwest.

Kiryandongo and Nakivale are two of Uganda’s oldest and most populous refugee settlements. The community of Kiryandongo was established in 1954 but formally instituted as a refugee settlement in 1990 by the Government of Uganda (GOU)^59^, while Nakivale was established in 1958 and formalized by the GOU in 1960^60^. Nakivale’s population of 176,720 predominantly consists of refugees from the DRC (66%) and Burundi (17.4%)^61,62^. Kiryandongo has 64,981 refugees, primarily from South Sudan (99%).

Access to the electricity grid is rare in both settlements, with 2.6% and 0% of households connected to the national grid for lighting in Nakivale and Kiryandongo, respectively^63^. In turn, a minority of residents have access to solar products. Kiryandongo has higher access to off-grid solar than Nakivale (21.5% vs.12.7%), with higher ownership of solar panels or electric inverters (26.7% vs. 5.9%). Data does not exist on the number of owners with certified products, but the share is likely significantly lower than the share of total solar owners. In both settlements, expansion has strained natural resources, with local woodland and bushland receding due to the need for fuel and farmland^64–67^.

### Context: barriers to solar adoption

To understand structural and behavioral barriers to access and adoption of solar products in Kiryandongo and Nakivale, we first conducted diagnostics in the settlements (see Supplementary Notes), which identified the following key constraints^18^:

First, the benefits of solar were not fully known or understood by refugees. In particular, given the high entry costs of purchasing solar products, the salience of energy savings over time and productive uses of solar products was low for this community. For example, 59% of respondents were not aware of possible productive uses of solar, such as extending opening hours due to light, playing music, or powering appliances^18^. The difficulty of assessing product benefits appeared to be due, in part, to low product availability and ownership in settlements, as well as limited bandwidth to calculate the possible savings or earnings associated with the impacts of solar products.

Second, trust in product quality was low in the target population, with 39% of respondents stating that the difficulty of distinguishing between good- and poor-quality solar products was a key reason for not purchasing solar products. Further, only 21% of respondents had heard of certified products and the purpose of international certification for distinguishing product quality. Information asymmetries and a high prevalence of low-quality and counterfeit products in the market meant that households did not purchase solar for fear products would break.

Third, low, unpredictable incomes and a lack of financing options for solar product purchases resulted in the sense that solar product ownership was out of reach for refugees. Approximately 63% of respondents in our study sample considered themselves unable to afford a solar product, and only 20% were saving for a solar product at baseline.

### Context: informal savings groups

As an alternative to formal banking in Uganda’s refugee settlements, VSLAs are collaborative, self-managed informal savings groups to which members contribute savings and that lend accumulated funds to members at relatively low interest rates (~5–10%). Members gather at an agreed-upon frequency, and each contributes at least a stipulated minimum amount throughout a savings cycle. One cycle usually lasts 10–12 months, after which the accumulated savings and the loan profits are distributed to the members.

VSLAs are common in both Kiryandongo and Nakivale settlements and often represent the key savings and loan vehicle for individuals in the absence of formal financial institutions. Evidence suggests that VSLAs are useful mechanisms for promoting saving toward income-generating activities^47^, buffer stock savings^68^, and other savings goals^48^. These groups are central to the implementation of this study’s intervention, given their important role in the financial and social lives of many refugees.

### Experimental design: intervention

We designed a behaviorally-informed intervention to address the identified barriers to adoption and used a cluster-Randomized Controlled Trial design (RCT) to test causal impacts on the pursuit of solar products. The design process was iterative, with several interventions first tested in an agile pre-pilot process (see Supplementary Notes). Final randomization took place at the VSLA level, with VSLAs randomly assigned to one of two conditions, a control group or a treatment group.

The VSLAs in the control group received no contact during the intervention period (between baseline and endline data collection). After the endline was completed, members in control VSLAs received the printed informational material distributed in treated VSLAs during the study period.

The intervention consisted of a behaviorally informed solar product information and savings session embedded within a VSLA meeting. The session was delivered by refugees and host community members living in and around the same settlement as the participants. It consisted of three parts: (1) salient information on the savings and productive benefits of solar products, (2) practical support to identify and access high-quality, certified solar products, and (3) public commitment to an attainable savings goal, whether for a solar product or another expense.

**Salient information: discussion of the possible savings and earnings benefits of solar products**. In the discussion, the VSLA members (“participants”) learned about the energy savings solar products can offer and their possible productive uses. The session was guided by printed flyers that used a refugee role model and specific examples to make the material more relatable. All materials were translated and delivered in the participants’ language. (See Supplementary Figs. 1–4 for materials in English.) In an attempt to avoid contamination from treatment to control, only one copy of each flyer used in the intervention was left at each VSLA after the intervention for reference within the group.

Note that the exact benefits of certified solar for a specific household depend on several variables: the amount a household currently spends to generate the power a device would provide, the household’s capacity to put aside a small amount of money for a sustained period for a device, and the total working life of the product purchased. We describe the calculation used for the savings flyer in Supplementary Fig. 1.

**Practical support: identifying certified devices and links to reputable local companies**. After discussing the benefits of solar, the session turned to practical considerations for choosing a solar product. The differences between certified and non-certified products were discussed, with pointers given for identifying certified devices. A catalog of certified solar products available locally was shared to inform participants about product types, prices, and access.

Information in the solar products catalog was gathered with support from local solar companies, whom the research team contacted by email and invited to submit relevant information about their products. The research team also visited the local offices of solar providers to collect information.

Participants were next informed about financing options for solar products. Options discussed included paying upfront, borrowing from the VSLA, or paying the solar company in installments (PAYGO). In all cases, the role of saving in a VSLA was emphasized.

**Goal-setting within the VSLA: establishing attainable savings goals for solar products or other purposes and publicly committing with VSLA support**. The session continued with a team game to draw attention to the group support and teamwork that can make VSLAs a powerful mechanism for saving. Participants were then encouraged to develop a savings goal, whether for a solar device or another expense. Goals were created using strategies informed by the WOOP (Wish, Outcome, Obstacle, Plan) method to ensure they were aspirational but also attainable^69^. Participants recorded the details of their goal on a simple goal-setting sheet, specifying (1) the purpose of their goal, (2) how much they planned to save at each VSLA meeting, and (3) the length of time they expected to need to achieve their goal.

The final part of the session involved an informal, verbal public commitment to each member’s savings goal in front of their VSLA cohort.

**Fig. 1:**
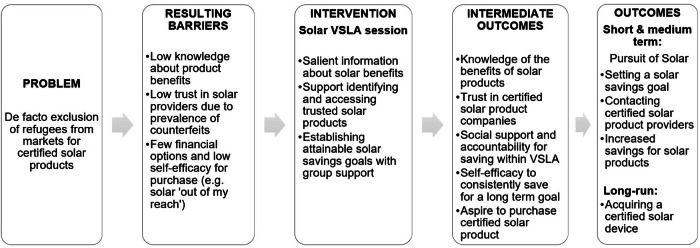
Theory of change for the adoption of solar home systems in refugee settlements.

The Theory of Change for the interventions described is outlined in Fig. 1. The first two boxes denote the key barriers identified through the study’s diagnostic work, the third box summarizes the interventions, and the final two boxes list the expected intermediate and final outcomes. In the “Results” section, we first estimate the impacts on the final outcomes and then use mediation analysis to study the effect of the intermediate outcomes on the final outcomes.

### Experimental design: sample and randomization

Since the intervention was delivered at the VSLA level, power calculations were implemented for a cluster-randomized trial design. The sample size was selected to allow the research team to calculate minimum effect sizes of 0.2 standard deviations (around 300 shillings, or $0.08) in the “weekly savings toward solar” outcome variable with 80% statistical power. This minimum detectable effect size is in line with the literature and allows for a small amount of attrition, given the relatively high mobility of our target population. For example, Salas ^48^ estimates an increase in savings of 35% due to public commitment in Colombia and 18–25% as a result of public labeling interventions. An effect size of 300 shillings would represent approximately a 35% increase from baseline levels in our sample.

A total of 312 VSLAs were identified through engagement with operating partners supporting the groups (e.g., BRAC, We Are Alight, Finnish Refugee Committee) and using snowball sampling through referrals from VSLA chairpersons or other members. VSLAs were eligible to participate if (1) they had at least eight members, (2) at least 50% of their members were refugees, (3) their group met at least once a month, and (4) at least half of members attended regular meetings, on average.

Group selection was followed by member sampling, in which eight participants were randomly selected from the group’s membership ledger. The first four members selected were to be a part of our sample, while the remaining four were to act as potential replacements if needed. VSLA members were eligible to participate if they (1) were a member of only one VSLA (the group they had been sampled from), a criterion used to minimize contamination between treatment and control, and (2) expressed interest in saving for a larger solar device than a simple lantern.

This study’s objectives were endorsed by UNHCR, and the work was conducted in close coordination with the government, UNHCR, and aid stakeholders. The study was implemented following the guiding principles of ethical research. Specifically, participation in the study was completely voluntary, and participants were asked to provide their written informed consent after being provided with details about the study protocol, data confidentiality, their freedom to withdraw at any time, and relevant contact details in the event questions, comments, or complaints arose. A witness was present for the consent for participants could not read themselves. Consent was collected at both baseline and endline surveys to ensure participants were reminded of the study details on confidentiality and accountability. In an effort to prioritize fairness between treatment and control groups, control VSLAs were provided with the informational material used for the intervention after endline data was collected.

Treatment was assigned using block-clustered-randomization at the VSLA level. Randomization was stratified using two variables: settlement (Nakivale (55%) or Kiryandongo (45%)) and the proportion of women in the savings group (75% or less (47% of VSLAs) or over 75% (53% of VSLAs)). We assigned 157 VSLA groups (628 individuals) to the treatment group and 155 VSLA groups (620 individuals) to the control.

### Experimental design: data and outcomes of interest

Baseline and endline surveys designed by the research team were carried out in person at the individual level. Data was collected from treatment and control participants before (September–December 2022) and after (May–June 2023) intervention implementation to compare changes in the two groups over the study period (see Supplementary Table 2 for more details about the project timeline).

The outcomes of interest measured in the surveys are described in Table 6 and mapped into the Theory of Change.

**Table 6:** Measured intervention outcomes

Outcome type		Variables	Description
Intermediate outcomes	Solar knowledge	• Knowledge of the benefits of solar products	Index combining three dummy variables about the benefits of solar targeted in the informational materials (saving money, making money, saving time). The index reflects respondents’ % score, depending on how many they remember.
		• Knowledge of how to identify certified products	Index combining five dummy variables about ways of identifying certified solar products (uniformed agents, branding, registration, contracts, warranties). The index reflects respondents’ % score, depending on how many they remember.
	Trust in solar companies	• Trust in solar companies selling certified products	Index combining four Likert variables about respondents’ trust in solar companies (trust to offer high-quality products, repayment flexibility, respectful treatment, repair, and replacement). The index reflects respondents’ average response on the 5-point Likert scale.
	Social support and accountability for saving	• Fellow VSLA members know your savings goals • Fellow VSLA members support your savings goals	Index combining the two Likert variables mentioned in the previous column. The index reflects respondents’ average response on the 5-point Likert scale.
	Self-efficacy/aspirations for solar	• Aspire to purchase solar	Dichotomous
		• Self-efficacy to save toward a solar goal	Index combining three Likert variables about respondents’ self-efficacy for saving (ability to stick to savings plans when there are unexpected expenses, ease of savings goals, and savings confidence). The index reflects respondents’ average response on the 5-point Likert scale.
Outcomes	Pursuit	• Have a solar savings goal	Dichotomous
		• Have a solar savings goal for a larger solar product	Dichotomous
		• Track saving	Dichotomous
		• Have contacted companies selling certified solar products	Dichotomous
		• Amount saving toward solar (per week)	Continuous
		• Expect to obtain a solar device next year	Continuous
	Ownership	• Own a solar device	Dichotomous
		• Own a larger solar device	Dichotomous
		• Have acquired a new solar device since March	Dichotomous

Outcome variables were collected in individual-level surveys designed by the research team for this purpose. The main analysis uses the data collected in the endline survey, implemented in 1203 households in May–June 2023.

Note that we measure outcomes in the short term in this report. Changes in outcomes such as solar acquisition (see Ownership above) likely take longer, since they can only occur once savings have accumulated. Thus, although we measure these for completeness, our focus is on outcomes related to Pursuit, such as savings toward solar devices. Our short-term focus allows us to model the establishment of a new savings habit and its mechanisms, a process we consider important for understanding adoption in this context. The outcomes are behavioral, separating this study from others (such as those mentioned in Supplementary Discussion 1) that use purchase intentions or willingness to pay measures as their outcomes, for instance. Despite this, a longer-term analysis that measures adoption outcomes when the time comes would be an important addition to this work.

Table 7 shows the balance between treatment and control on VSLA characteristics, and Supplementary Tables 3 and 4 display balance at the individual level for demographic and outcome variables, respectively. We use difference-in-means tests and Romano–Wolf multiple hypotheses testing algorithms for this analysis.

**Table 7:** Balance table for VSLA characteristics

Variable	Control mean (SD) *N* = *155*	Treatment mean (SD) *N* = *157*	C-T (SE)
Number of members	20.28	20.11	−0.17
	(10.59)	(14.10)	(1.31)
Number of male members	6.11	6.16	0.05
	(5.91)	(9.19)	(0.70)
Number of female members	14.17	13.95	−0.22
	(7.73)	(8.48)	(0.84)
VSLA share price^1^	3577.74	3542.04	−40.17
	(7972.94)	(6295.81)	(811.86)
VSLA meeting at least once a week	0.88	0.85	−0.04
	(0.32)	(0.36)	(0.04)

The table includes data from 280 baseline VSLAs collected for the initial VSLA scoping exercise during sampling. Balance tests were conducted using t-tests, with Romano–Wolf corrections for multiple hypothesis testing later run (but not reported here). Column 2 reports the mean of control VSLAs and column 3 the mean of treatment VSLAs. Standard deviations are presented below in parentheses. Column 4 displays the difference between the two means, with standard errors displayed in parenthesis. Asterisks denote a statistically significant difference at the 1% ***, 5% **, or 10% * levels.

^1^Normalized for meeting frequency to a weekly share price.

At the VSLA level, our sample is balanced across treatment and control groups, with no significant differences in demographic characteristics and outcomes of interest before intervention rollout. For the individual-level demographics, the sample is balanced, except for whether the respondent has primary-level education and on-farm wage employment. These differences are not significant after correcting for multiple hypothesis testing, however. Finally, baseline measures of the outcome variables are balanced.

### Implementation: baseline survey and replacements

At baseline, we interviewed 1041 respondents (533 treatment and 508 control) from 280 VSLAs (142 treatment and 138 control) in our sampling frame of 312 VSLA groups. Data was not collected from all VSLAs due to time constraints for the intervention’s rollout and the need to start implementation as close to the start of the savings cycle as possible. Due to this limited baseline sample (~90% of VSLAs), the analysis below uses only endline data, where data collection was attempted in all 312 groups. Baseline data is used to conduct robustness tests included in the supplementary materials (Supplementary Methods).

Intervention rollout and endline survey collection were still attempted in all 312 VSLAs in our sample, including all 157 treatment VSLAs originally assigned to the treatment group before baseline collection.

To mitigate the risk of attrition during the study, the intervention was timed to maximize exposure to a single VSLA cycle. Sampling thus occurred toward the end of the previous VSLA cycle to allow the team to roll out the intervention toward the beginning of a new cycle. Though few, this method necessitated some replacements before the intervention began, since VSLA membership can shift slightly between savings cycles. However, the team preferred to make replacements before the intervention rollout than to lose participants during or after the rollout. The replacement process is described below.

As mentioned, sampled individuals who withdrew from the study before the intervention rollout were replaced by a different member of their VSLA. Replacements occurred if individuals declined consent for intervention participation, migrated, or left their VSLA before intervention activities commenced. The process was mirrored for control VSLA members before the endline survey, with replacements for those who had migrated or left their VSLA before March 2023 (the start of the intervention in treatment). Note that sampled members who provided consent and remained in their VSLA but did not attend the treatment session remained in the study sample.

In total, 52 individuals were replaced before the intervention rollout, 36 in the treatment and 16 in the control. Our final sample consists of 1248 individuals (628 in treatment VLSA groups and 620 in control VSLA groups).

Though replacements may differ slightly from the original selected sample, we consider this process to be a necessary part of sampling when conducting research with transient populations such as refugees, and with VSLAs, where membership can alter between savings cycles. We consider the results presented below to be a fair representation of the impacts of the intervention in Nakivale and Kiryandongo for the population that could participate.

Note that we cannot always distinguish in the data between the 52 replacements and the 185 original sampled members for whom we did not collect baseline data. The empirical strategy (see next section) controls for the individual being in either of these groups in case of differences.

### Implementation: compliance and attrition

Of the 157 VSLAs assigned to treatment, the intervention was rolled out successfully in 143 VLSAs. Rollout was not possible in 14 VSLAs due either to an inability to locate the group (4), dissolved groups (4), an insufficient number of members for regular VSLA activities (3), or withdrawal from the study (1). For this reason, the effects estimated below represent an intention-to-treat (ITT) analysis, and thus likely represent a lower bound of impact. As mentioned, all individuals selected for treatment and not replaced were included in the endline data collection regardless of their treatment compliance.

In total, the intervention was successfully delivered to 537 individuals across 143 VSLAs (437 (82%) of these with baselines). This number corresponds to 86% of the treatment sample after replacements (see Table 8):

**Table 8:** Intervention compliance

	VSLAs	Individuals
Assigned to Treatment	157	628
Complied (Treated)	143	537
% Compliance	91%	86%

The table displays the number of VSLAs and individuals assigned to treatment (row 1) and the number who attended treatment (row 2). Compliance is calculated in row 3. Note there are 71 treatment respondents whose compliance cannot be ascertained objectively (with attendance data from the session), as it is based on self-reports. Of these, 35 (49%) report attending the session.

We attempted to collect endline data for all sampled members across the 312 VSLAs and 1248 individual members in the final sampling frame. Treatment VSLAs were interviewed at least four weeks after the rollout of the intervention, except for nine groups for which we had to conduct the endline survey before the four-week period ended due to delays with intervention implementation. The endline survey was administered in a matched pair of treatment and control VSLAs from the same randomization blocks to avoid timing biases. We successfully collected 1203 endline responses (604 from treatment VSLAs and 599 from control VSLAs) from 309 VSLAs (155 from the treatment and 154 from the control). Attrition was therefore 3.6% (see Table 9), which was less than our anticipated level of attrition in our power analysis.

**Table 9:** Endline attrition

	VSLAs			Individuals		
	Treatment	Control	Total	Treatment	Control	Total
Total Sample	157	155	312	628	620	1248
Endline	155	154	309	604	599	1203
% Attrition	1%	1%	1%	4%	3%	3.6%

The table displays the number of VSLAs and individuals in the treatment and the control group who should have received (row 1) and who did receive (row 2) an endline survey. Attrition is calculated in row 3.

### Empirical strategy: ordinary least squares (OLS)

Due to the randomized design, the study’s primary method of estimation is a simple linear regression model comparing differences between treatment and control VSLAs at endline.

1 $$Yi,{vsla}=\beta 0+\beta_ 1T{vsla}+\beta_ 2Ri,{vsla}+{\boldsymbol{\gamma }}{\bf{C}}v{sla}+{\boldsymbol{\delta }}{\bf{X}}i,v{sla}+\epsilon i,{vsla}$$

$Yi,{vsla}$ is a given outcome variable of interest at endline.

$T{vsla}$ is a dummy variable indicating whether the individual’s VSLA was assigned to the treatment group (equal to 1, otherwise 0).

$Ri,v{sla}$ is a dummy variable indicating if an individual is a replacement or lacks baseline data (equal to 1, otherwise 0).

${\bf{C}}v{sla}$ are control variables at the VSLA level (a settlement dummy, a dummy for VSLAs that meet at least weekly, the number of members in the VSLA, and the VSLA’s share price, normalized to weekly).

${\bf{X}}i,v{sla}$ are control variables at the individual level (dummy for at least primary-level education).

$\epsilon i,{vsla}$ is the error term, clustered at the VSLA level.

The analysis is an intent-to-treat, given imperfect intervention compliance. All OLS regressions are robust to specifications for binary dependent variables where relevant (see Supplementary Tables 5 and 6). However, we favor OLS due to the ease of interpretation as well as its reliability for estimating the effects of binary treatments on binary outcomes^70^.

### Empirical strategy: Tobit regressions

Some of the study’s outcomes are conditional on other outcomes. For instance, the amount of shillings saved toward a solar product is conditional on an individual saving for that product. We use specifications suitable for censored variables for these outcomes since they are censored at zero for non-savers. Tobit regressions are used to estimate the impact of treatment on the censored variable rather than on the latent variable (as in Samantaraya & Patra^71^). The coefficients reported are the average marginal effects for these censored outcome variables.

### Empirical strategy: ANCOVA and difference-in-differences regressions

For robustness, we compare the analysis above with one that controls for baseline values of the outcome variables (ANCOVA)^72^ and to a difference-in-differences (DID) specification using the baseline and endline data. Since we have some missing baseline surveys for participants, our preferred specification is the single-wave OLS analysis described above. However, ANCOVA and difference-in-differences are used as robustness tests in the Supplementary Methods section (Supplementary Tables 7–9 for ANCOVA and 10–12 for DID).

The ANCOVA analysis is specified below. Note that, to maximize the use of the sample, individuals without baseline surveys (for whom $Ri,{vsla}=$ 1) are still included in the specification by setting the baseline level of their outcome variables to zero and controlling for the dummy identifying them as individuals without baselines^73^.

2 $$\begin{array}{l}Yi,{vsla},t=1=\beta_ 0+\beta_ 1T{vsla}+\beta_ 2Ri,{vsla}+\sigma_ 1Yi,{vsla},t=0\\\qquad\qquad\quad+\,{\boldsymbol{\gamma }}{\bf{C}}{vsla}+{\boldsymbol{\delta }}{\bf{X}}i,{vsla}+\epsilon i,{vsla}\end{array}$$

The DID analysis is specified, as displayed below, by regressing a given outcome of interest on a dummy for treatment status at the VSLA level, a dummy for being in the second period (at endline: $Et$), and the interaction of the two variables.

3 $$\,Yi,{vsla}=\beta_ 0+\beta_ 1T{vsla}+\beta_ 2Et+\beta_ 3\left(Et* T{vsla}\right)+\epsilon i,{vsla}$$

The results presented below are robust to the ANCOVA and DID specifications presented in Supplementary Methods.

### Mediation analysis

In this section, we evaluate whether any of the impacts reported were mediated through the intermediate outcomes outlined in the Theory of Change. This analysis allows us to assess whether we see impacts manifest through the barriers we identified and to ask which, if any, were most relevant. Isolating the mechanisms for effects causally would require multiple treatment arms with the assignment of different subsets of the treatment to each arm. Multiple arms were beyond the remit of this study, but we can explore the degree to which impacts occurred through mediators using seemingly unrelated regression models^74^. Though the mediation estimates presented below are not causal and therefore should be interpreted with some caution, these analyses can still be very informative, especially when accompanied by a clear theoretical model and if confounders are adequately controlled for.

Seemingly unrelated regression analysis allows us to investigate relationships between regressions for each stage in the Theory of Change. In particular, it assesses the relationship between the effect of the treatment on the mediators, the effect of the mediators on the outcome, and the effect of the treatment itself on the outcome while controlling for the possible mediators. The effect of the treatment on the outcome is then decomposed into an indirect effect (through the mediator) and a direct effect (straight from the treatment to the outcome). Indirect effects (or mediated effects) are what we evaluate to ascertain whether there has been mediation. The proportion mediated is calculated by dividing the mediated effect by the total effect.

We evaluate the following mediators (also presented in Fig. 1). All mediators are indices except for the aspirations variable, which is dichotomous (see Supplementary Table 1 for details):

- Solar knowledge: knowledge of the benefits of solar products and of how to identify certified products (these indices are combined due to multi-collinearity if entered separately in the analysis).
- Trust: trust in companies selling certified solar products locally.
- VSLA support: a respondent’s perception of being supported and held accountable by their VSLA.
- Aspirations: an aspiration to purchase a solar product in the coming year. (Note that since our sample was selected on similar criteria to this—namely, being interested in saving toward a larger solar device, rather than wanting to purchase one in the coming year—our sample likely has higher aspirations for solar than a randomly selected sample would. Despite this, we still see positive impacts of the intervention on solar aspirations and are thus interested in this variable as a potential mediator).
- Self-efficacy: a respondent’s self-belief in their ability to consistently save for a long-term goal such as solar products.

The outcomes for which we consider possible mediation are three of the key impacts presented in the “Results” section:

- Contacting a solar company,
- Having a solar product savings goal,
- Weekly solar product savings with a respondent’s VSLA.

Since we do not find impacts on product ownership in our short study, this outcome is not included in the mediation analysis. However, Supplementary Discussion 1 explores the links between the outcomes we do detect and this ultimate outcome, considering existing literature that examines these links.

For the first two outcomes listed above, we employ the same OLS regression specifications, clustering of standard errors, and control variables, as described in the Empirical Strategy. The command “sureg” is used in Stata, adapted for clustered standard errors using “suregr.” Bootstrapping is then used for the mediation analysis itself, in which bias-corrected and accelerated (Bca) standard errors and confidence intervals are calculated. The results are presented in the first two panels of Table 5.

Due to the need for regressions appropriate for a censored dependent variable, we do not employ seemingly unrelated regression analysis for the solar savings outcome. Rather, we use a more descriptive approach, running regressions separately for the treatment on the mediators (OLS), the mediators on the outcome (Tobit), and the treatment itself on the outcome while controlling for the possible mediators (Tobit). As a result, we focus more on the size and direction of links between variables than on the significance and proportion of mediation for this variable. This analysis is displayed in the third panel of Table 5.

## Supplementary information

Supplementary Material

## Data Availability

The anonymized versions of the datasets generated for this study are available upon request. They will be posted within two years of publication at https://microdata.worldbank.org/index.php/home.

## Code availability

Computer code available upon request.
