# Supplementary material for: Behavioral savings sessions increase the pursuit of solar products among refugees in Uganda
Source: NPJ Clim Action. 2025 Mar 29;4(1):32. doi: 10.1038/s44168-025-00212-x (PMC11954683; doi:10.1038/s44168-025-00212-x)
Supplement: Supplementary file 1 — Supplementary Material [file 44168_2025_212_MOESM1_ESM.pdf]

Supplementary Material

Supplementary Figures

Supplementary Figure 1: Flyer 1- Benefits of Solar

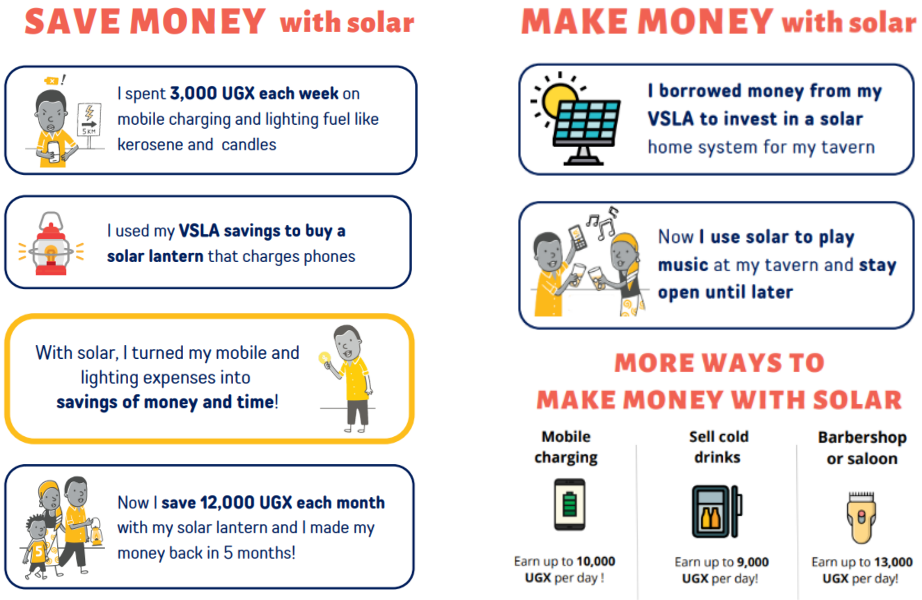

Notes: A household spending 3,000 UGX ($0.81) per week on lighting and charging (a conservative calculation based on data collected in spending diaries collected by Low-Income Financial Transformation in Kiryandongo and Nakivale in 2022) would recover the cost of a certified solar lantern with charging capacity (around 65,000 UGX, or $17.50) in 5 months (22 weeks). It would take 33 weeks (~8 months) to purchase this device while saving 2,000 UGX ($0.54) weekly with the VSLA (also a conservative estimate, based how much extra money refugees had stated they would be able to put aside on top of their regular savings in the data mentioned above). However, this would generate savings for the working life of the product (which has a two-year warranty, so a minimum of two years, or minimum savings of 104*3000=312,000 UGX, or $57.50). Savings would exceed the cost of the product after 5 months (22*3000=66,000 UGX, or ~$18).

Supplementary Figure 2: Flyer 2- Identifying Certified Products

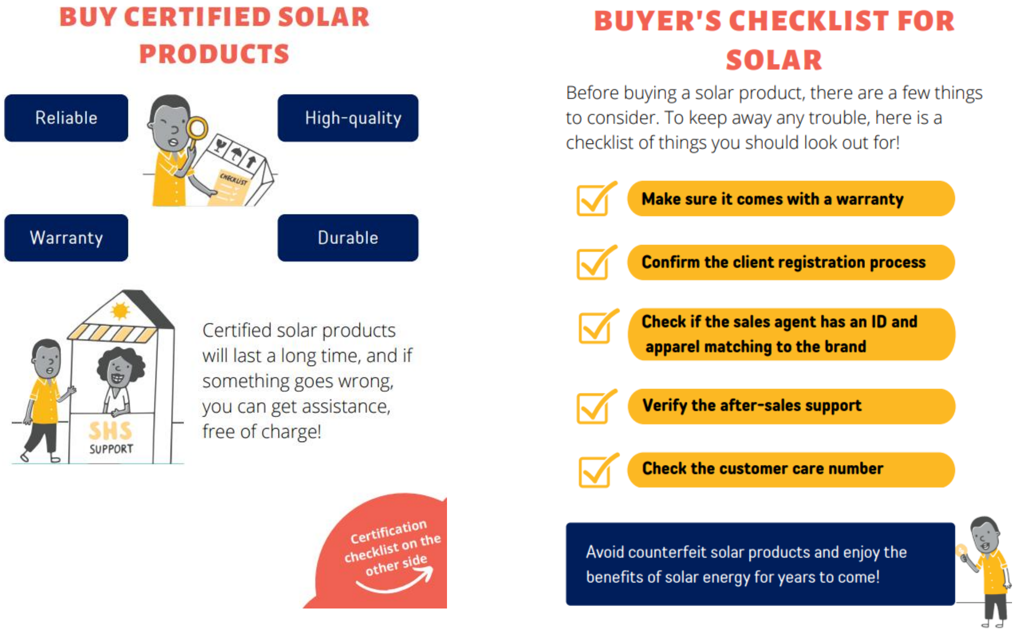

Supplementary Figure 3: Flyer 3- Solar Catalogue of Locally Available Certified Products

	Product	Description
SMALL	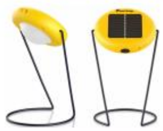	Light: 1 light<br>Phone charging: No<br>Panel: Mini, built in<br>Warranty: 2 years<br>**Brand: Sun King**<br>**Price: UGX 39,000**
SMALL	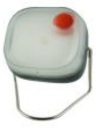	Light: 1 light<br>Phone charging: No<br>Panel: Mini, built in<br>Warranty: 1 year<br>**Brand: d.light**<br>**Price: UGX 20,000**
MEDIUM	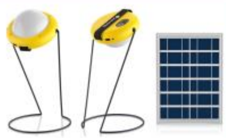	Light: 1 light<br>Phone charging: Yes<br>Panel: Small sized<br>Warranty: 2 years<br>**Brand: Sun King**<br>**Price: UGX 163,000**
MEDIUM	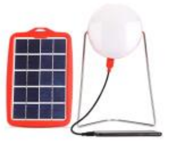	Light: 1 light<br>Phone charging: Yes<br>Panel: Small sized<br>Warranty: 2 years<br>**Brand: d.light**<br>**Price: UGX 65,000**

For more information, please call:

Sun King: 0800-203-070 (toll free)
Engie: 0800-202-933 (toll free from 7am-6pm, excluding public holidays)
D.Light: 078-602-31-36
Solar Today: 078-660-42-35 (Nakivale-Mbarara)
Fres Uganda: 078-757-45-94 (Nakivale-Mbarara)
Power Trust: 075-283-78-48 (Kiryandongo-Bweyale)

	Product	Description
LARGE	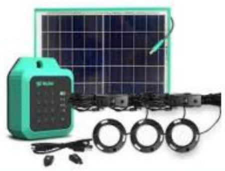	Light: 3 lights<br>Phone charging: Yes<br>Panel: medium sized<br>Warranty: 3 years<br>**Brand: Engie**<br>**Price: UGX 439,000**
LARGE	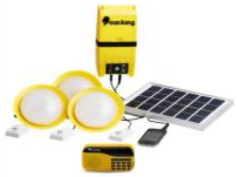	Light: 3 lights<br>Phone charging: Yes<br>Panel: medium sized<br>Warranty: 2 years<br>**Brand: Sun King**<br>**Price: UGX 439,000**
LARGE with accessories	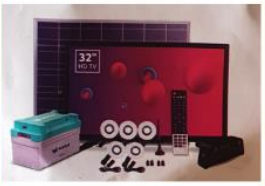	Light: 5 lights<br>Phone charging: Yes<br>Panel: 120W and 400Wh battery<br>Accessories: 32" TV and speaker<br>Warranty: 3 years for panel and device, 2 years for accessories<br>**Brand: Engie**<br>**Price: UGX 3,200,000**
LARGE with accessories	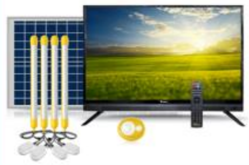	Light: 4 lights<br>Phone charging: Yes<br>Panel: 50W and 140Wh battery<br>Accessories: 24" TV<br>Warranty: 2 year<br>**Brand: Sun King**<br>**Price: UGX 2,278,000**

Supplementary Figure 4: Flyer 4- Goal-Setting Sheet

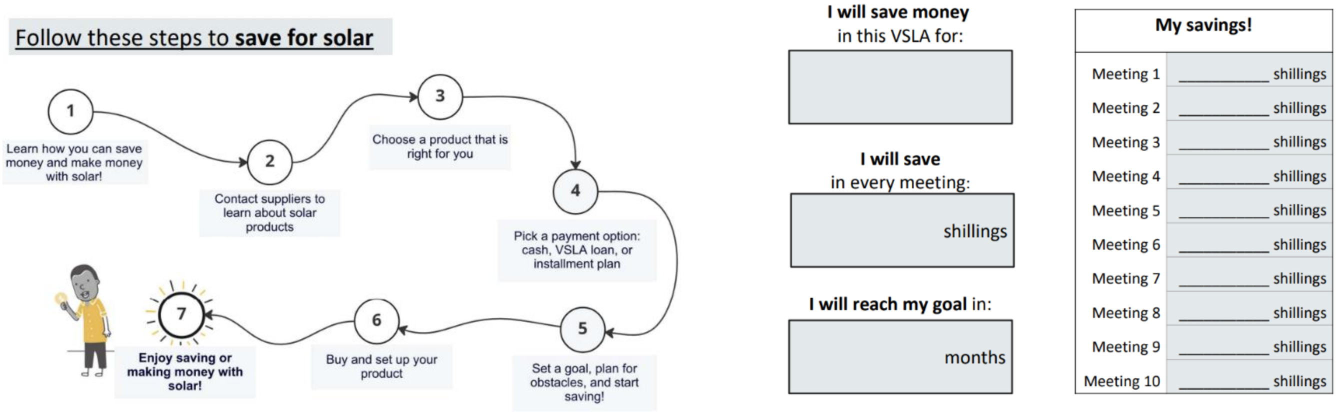

Supplementary Figure 5. Mediation analysis of treatment impact on likelihood of contacting a solar company.

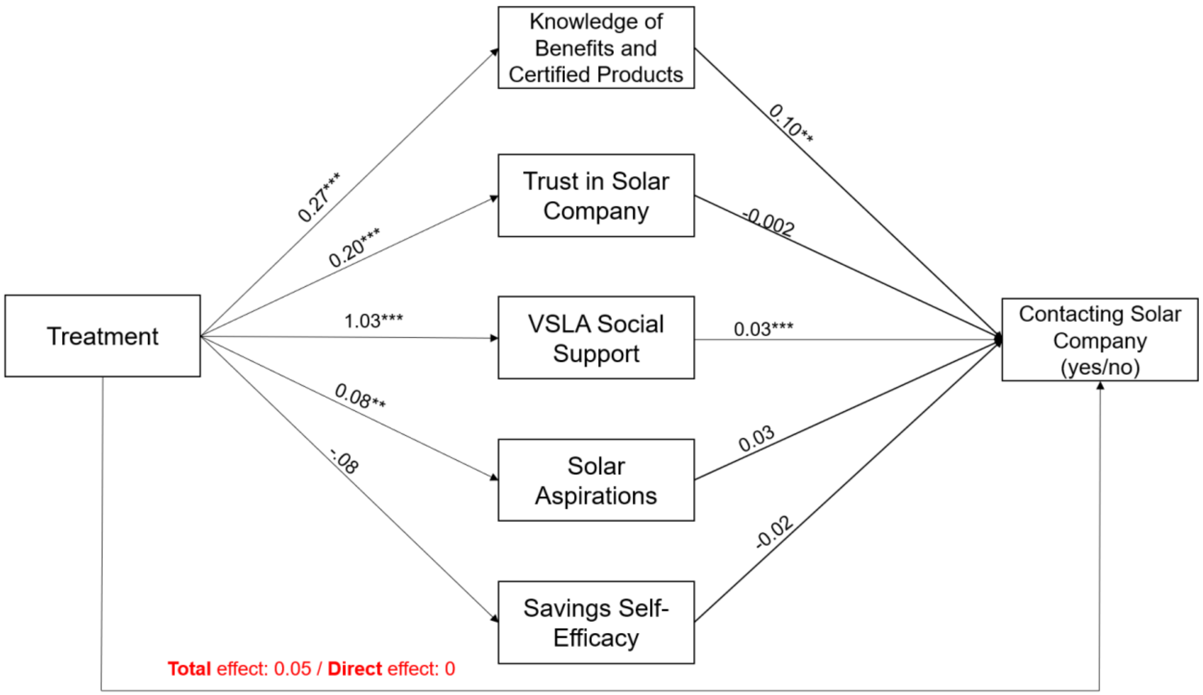

Notes: The figure displays the values estimated in the mediation analysis presented in the main paper. Coefficients are non-standardized. Arrows on the left of the diagram represent the impact of treatment on the mediators, while those on the right represent the impact of mediators on the outcome over and above their effect through treatment. The arrow at the bottom of the diagram denotes the direct effect of treatment on the outcome of interest. Asterisks denote a statistically significant difference at the 1% ***, 5% **, or 10% * levels.

Supplementary Figure 6. Mediation analysis of treatment impact on likelihood of having a solar savings goal.

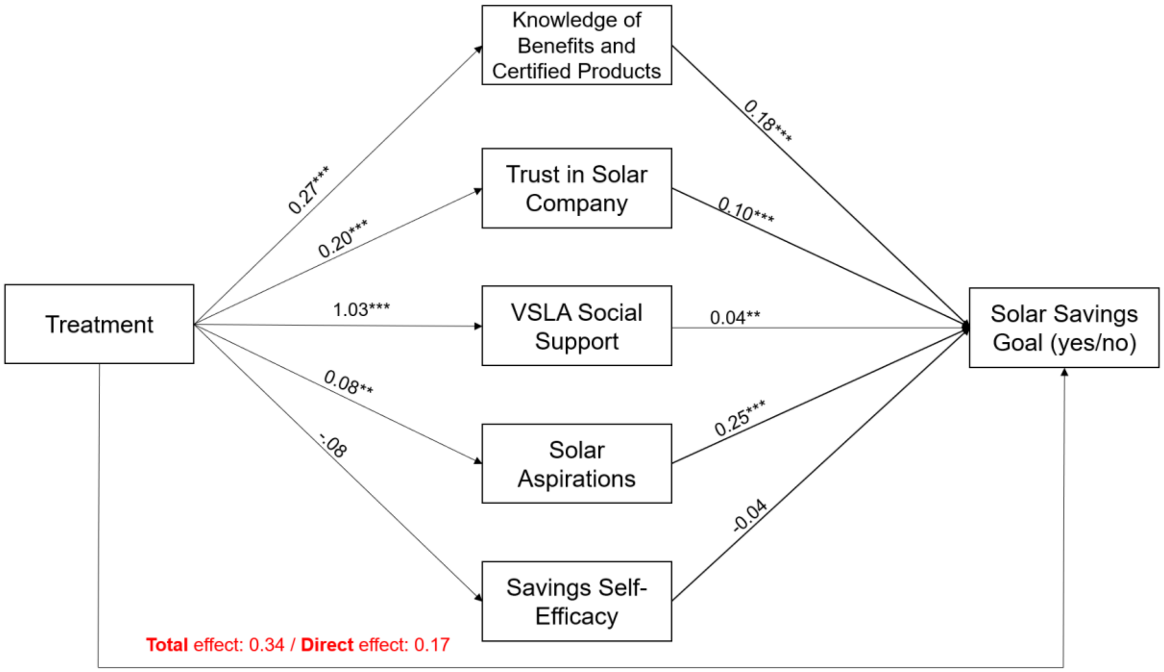

Notes: The figure displays the values estimated in the mediation analysis presented in the main paper. Coefficients are non-standardized. Arrows on the left of the diagram represent the impact of treatment on the mediators, while those on the right represent the impact of mediators on the outcome over and above their effect through treatment. The arrow at the bottom of the diagram denotes the direct effect of treatment on the outcome of interest. Asterisks denote a statistically significant difference at the 1% ***, 5% **, or 10% * levels.

Supplementary Figure 7: Mediation analysis of treatment impact on weekly solar savings with the VSLA.

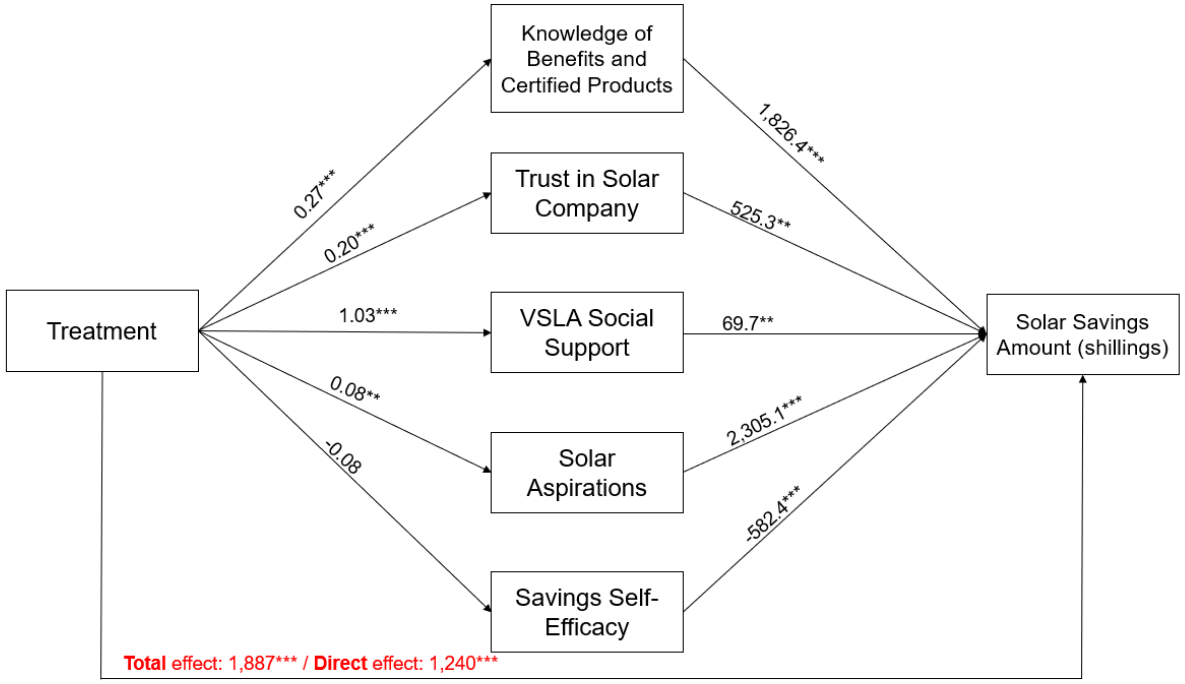

Notes: The figure displays the values estimated in the mediation analysis presented in the main paper. Coefficients are non-standardized. Note that the (descriptive) analysis for this outcome variable was carried out by running regressions separately for each stage of the Theory of Change. Arrows on the left of the diagram represent the impact of treatment on the mediators (OLS), while those on the right represent the impact of mediators on the outcome, over and above their effect through treatment (Tobit). The arrow at the bottom of the diagram denotes the direct effect of treatment on the outcome of interest. Asterisks denote a statistically significant difference at the 1% ***, 5% **, or 10% * levels.

Supplementary Notes

Diagnostic

During initial work in 2019 and 2020, we sought to understand barriers to access and adoption of solar products by carrying out a qualitative diagnostic study. This consisted of a desk review of literature pertaining to barriers to the adoption and sustained use of energy-efficient technologies, as well as field research with the target population in the Kyangwali and Rhino Camp settlements. The two settlements were selected due to their different geographic locations, demographics, and characteristics in the hope that the fieldwork would offer a relatively broad perspective, despite the small sample size. The fieldwork included households with and without solar home systems, community and VSLA groups, market and shop visits, and visits to public institutions, such as schools and health centers. Around 130 people took part in interviews as part of this diagnostic fieldwork, which included semi-structured interviews, focus groups, and marketplace and energy kiosk observations.

The diagnostic process allowed us to better understand the context of solar use in Ugandan refugee settlements. The team identified a common “user journey” for the adoption of solar products and was able to pinpoint possible barriers at different stages in this process. Using these findings, we designed a range of possible interventions to target barriers to solar adoption, and particularly the adoption of larger solar devices, such as solar home systems. As mentioned in the paper, all these interventions were created with the following aims:

1. To emphasize the possible savings and earnings benefits of solar products.
2. To inform participants about how to identify high-quality, certified products.
3. To explore the social and risk-reduction benefits of leveraging village savings and lending groups as a sustainable method of supporting savings or loans for certified solar.

Pre-Pilot

To hone our intervention design, a pre-pilot study was carried out in Kiryandongo and Nakivale refugee settlements in August and September 2022. These are the same areas in which the final study was also carried out, but pre-pilot activities were performed in different VSLAs than those sampled for the final study. In the pre-pilot exercise, three different activities were tested by at least 100 respondents each (see Supplementary Table 1 for precise samples and for the channels that each intervention was designed to target, and the intervention materials below):

1. The first component A/B-tested informational flyers (maximum two flyers per person) to gauge what type of information and flyer design was most engaging and informative to recipients. Information about product benefits, access, and identifying certified products was covered. This component also sought basic information from participants to check that the qualitative findings from the pre-pilot also applied in the pilot settlements. The questionnaire asked about the extent of knowledge of solar benefits, local solar options, certified products, and barriers to access for respondents.
2. The second component tested several interventions to boost socio-emotional skills for saving. We piloted a public commitment activity, a WOOP (Wish, Outcome, Obstacle, Plan) exercise, a habit formation activity, and a role model video. We also trialed a team-building game involving balloons to emphasize the importance of group support for individual and collective goals.
3. In the final component, we collected detailed information about participants’ energy spending and saving with the goal of offering personalized support in selecting a solar product and payment method suited to their circumstances.

Supplementary Table 1: Pre-Pilot Breakdown

Component	Intervention	Targeted Channels	N (All)	N (Nakivale)	N (Kiryandongo)
1	A/B testing of flyers	Lack of salience of solar benefits.<br><br>Low trust in solar providers. Market information asymmetries.	122	55 (45%)	67 (55%)
2	Public commitment intervention, WOOP, role model video, habit formation exercise, balloon game	Lack of financing options for acquiring solar and feeling solar products are out of reach for refugees.	186	62 (33%)	124 (67%)
3	Personalized energy expenditure tracking and personalized solar suggestions.	Lack of salience of solar benefits.<br><br>Lack of financing options for acquiring solar and feeling solar products are out of reach for refugees.	105	50 (48%)	55 (52%)

Component 1

Results from Component 1 suggested that different information was useful for different types of participants. In qualitative feedback from respondents, it seemed that information about energy savings was most appealing to slightly poorer participants, while information about making money from solar tended to resonate more with wealthier respondents and business owners. Given the range of backgrounds and geographies in our sample, we decided to keep information about both saving and making money. Finally, the information provided about certified products was new to participants and considered helpful (59% said they learnt a lot from it).

During the pre-pilot, many respondents requested more precise information about the products available in their area, their cost, and where they could be accessed. For this reason, we designed a solar catalogue of vendors selling certified products locally, which was implemented in our final intervention.

Component 2

The role model video was popular in this component, though the content we tested in the pre-pilot was not directly suited to our setting. Since generating a more relevant video of our own was not feasible for the study timeline, however, we decided to focus on other popular interventions from component 2.

Since 86% of participants said they needed to commit themselves to achieve their savings goals, the team moved forward with the public commitment intervention, incorporating WOOP elements

to ensure goals were attainable. We also opted to keep the team-building balloon game, since it was received very enthusiastically by participants and was perceived in subsequent discussions to have delivered a clear message about teamwork.

The habit-formation intervention was challenging to implement in the context of the settlements where we were working, given the need to identify a specific behavior or cue to link savings to. Furthermore, the intervention was considered less crucial, since savings habits in the community tended to already be strong, as long as savers could commit to their goals.

Component 3

Component 3 was a little too complicated for large-scale rollout in its initial format, taking a long time to implement and with material often not retained by participants. However, tracking energy spending was a popular component 3 activity, since it helped participants envisage what they could save if they owned a solar product. Given this, we included a clear, relatable example of energy savings in the intervention flyers (component 1) but moved away from detailed energy tracking to maximize the scalability and simplicity of our intervention.

Supplementary Tables

Supplementary Table 2: Timeline of Activities

Activity	Dates
**Diagnostics**	July 2019-Feb 2021
**Pre-pilot**	August 2022
**Sampling**	August-September 2022
**Baseline survey**	October 2022-January 2023
**Randomization and balance checks**	February 2023
**Intervention rollout**	March-June 2023
**Endline survey**	May-June 2023

Supplementary Table 3: Individual Level Demographic Variables for Balance at Baseline

Variable	Control Mean (SD)	Treatment Mean (SD)	C-T (SE)	n
Poverty Index: Lowest Quintile	0.196	0.190	-0.006	922
	(0.397)	(0.393)	(0.033)	
Poverty Index: Quintile 2	0.209	0.208	0.002	922
	(0.407)	(0.406)	(0.031)	
Poverty Index: Quintile 3	0.176	0.214	0.039	922
	(0.382)	(0.411)	(0.027)	
Poverty Index: Quintile 4	0.228	0.182	-0.046	922
	(0.420)	(0.386)	(0.031)	
Poverty Index: Highest Quintile	0.191	0.206	0.011	922
	(0.394)	(0.405)	(0.031)	
Poverty Index	11.566	11.584	0.012	922
	(0.735)	(0.747)	(0.065)	
Total Savings	157765.109	183309.844	21,982.994	750
	(246708.063)	(309749.781)	(26,678.039)	
Total Savings last week	6,545.116	6,523.092	-61.159	1,023
	(7,753.621)	(8,721.529)	(675.608)	
No education	0.187	0.254	0.077**	1,032
	(0.390)	(0.436)	(0.030)	
Completed primary and higher	0.484	0.396	-0.096***	1,032
	(0.500)	(0.489)	(0.036)	
Completed secondary and higher	0.071	0.061	-0.015	1,032
	(0.258)	(0.239)	(0.019)	
Some university or higher	0.026	0.032	0.004	1,032
	(0.159)	(0.177)	(0.012)	
Age of respondent	37.087	36.559	-0.516	1,041

	(11.337)	(12.478)	(0.907)	
Number of household members	7.254	7.343	0.164	1,041
	(3.526)	(4.578)	(0.358)	
Years in Uganda	13.469	10.797	-2.512	1,041
	(37.373)	(7.494)	(1.693)	
Years in Settlement	10.894	10.227	-0.630	1,041
	(7.299)	(7.036)	(0.660)	
Gender of respondent	0.715	0.709	0.018	1,041
	(0.452)	(0.455)	(0.024)	
Respondent is Household Head	0.687	0.692	0.008	1,041
	(0.464)	(0.462)	(0.031)	
Uganda Nationality	0.002	0.006	0.004	1,041
	(0.044)	(0.075)	(0.004)	
South Sudan Nationality	0.490	0.448	-0.000	1,041
	(0.500)	(0.498)	(0.012)	
DRC Nationality	0.323	0.326	-0.025	1,041
	(0.468)	(0.469)	(0.035)	
Burundi Nationality	0.112	0.143	0.020	1,041
	(0.316)	(0.350)	(0.032)	
Somalia Nationality	0.000	0.000	0.000	1,041
	(0.000)	(0.000)	(0.000)	
Rwanda Nationality	0.061	0.066	0.002	1,041
	(0.240)	(0.248)	(0.019)	
Tanzania Nationality	0.000	0.002	0.002	1,041
	(0.000)	(0.043)	(0.002)	
Ethiopia Nationality	0.000	0.000	0.000	1,041
	(0.000)	(0.000)	(0.000)	
Kenya Nationality	0.012	0.002	-0.009	1,041
	(0.108)	(0.043)	(0.008)	
Sudan Nationality	0.000	0.008	0.008	1,041
	(0.000)	(0.086)	(0.007)	
Main source of Household Income: Non-Farm Enterprise owner/Entrepreneur	0.102	0.086	-0.013	1,041
	(0.303)	(0.281)	(0.022)	
Main source of Household Income: Wage Employee, Non-Farm	0.022	0.026	0.003	1,041
	(0.146)	(0.160)	(0.009)	
Main source of Household Income: Wage Employee, Farm	0.065	0.038	-0.027*	1,041
	(0.247)	(0.190)	(0.016)	
Main source of Household Income: Self-Employed Non-Farm-Independent contractor, technician, professional, etc.	0.045	0.036	-0.010	1,041
	(0.208)	(0.186)	(0.015)	
Main source of Household Income: Self-Employed Agriculture/Livestock	0.177	0.133	-0.040	1,041
	(0.382)	(0.340)	(0.032)	
	0.031	0.026	-0.005	1,041

Main source of Household Income: Property income	(0.175)	(0.160)	(0.014)	
Main source of Household Income: Casual/Day Laborer	0.323	0.379	0.048	1,041
	(0.468)	(0.486)	(0.039)	
Main source of Household Income: Transfers (pension, allowances, social security benefits)	0.014	0.008	-0.006	1,041
	(0.117)	(0.086)	(0.007)	
Main source of Household Income: International/foreign remittances	0.016	0.015	0.000	1,041
	(0.125)	(0.122)	(0.007)	
Main source of Household Income: Organizational support from WFP	0.866	0.841	-0.018	1,041
	(0.341)	(0.366)	(0.035)	
Main source of Household Income: Organizational support from UN	0.116	0.148	0.040	1,041
	(0.321)	(0.356)	(0.036)	
Main source of Household Income: Organizational support from UNICEF	0.000	0.004	0.004	1,041
	(0.000)	(0.061)	(0.003)	
Main source of Household Income: Organizational support from other/NGO	0.075	0.096	0.027	1,041
	(0.263)	(0.294)	(0.029)	
Main source of Household Income: Other	0.098	0.113	0.006	1,041
	(0.298)	(0.316)	(0.028)	
HH has business or enterprise	0.293	0.248	-0.053	1,041
	(0.456)	(0.432)	(0.034)	
Respondent responsible for HH business decision	0.758	0.737	-0.015	282
	(0.430)	(0.442)	(0.055)	
HH business revenue	187701.906	211448.844	31,467.289	195
	(406760.406)	(393664.969)	(59,503.125)	
Electricity supply as enterprise bottleneck	1.174	1.113	-0.094	282
	(3.745)	(3.978)	(0.532)	
**N**	**508**	**533**	**1,041**	

Notes: The table includes data from 1,041 baseline individuals collected during the baseline survey. Balance tests were conducted using t-tests, with Romano Wolf corrections for multiple hypothesis testing run later (but not reported here). Column 2 reports the mean of control VSLAs and column 3 the mean of treatment VSLAs. Standard deviations are presented below in parenthesis. Column 3 displays the difference between the two means, with standard errors displayed in parenthesis. Asterisks denote a statistically significant difference at the 1% ***, 5% **, or 10% * levels.

Supplementary Table 4: Individual Level Outcome Variables for Balance at Baseline

Variables	Control Mean/(SE)	Treatment Mean/(SE)	C-T Mean/Sig	N
**Intermediate Outcomes**				
Knowledge of Targeted Solar Benefits: Index: Baseline	0.488	0.515	-0.023	1,041
	(0.301)	(0.300)		
Knowledge of Certified Products: Index: Baseline	0.087	0.080	-0.006	1,041
	(0.217)	(0.203)		
Aspire to purchase solar this year: Baseline*	0.941	0.904	0.021	1,041
	(0.236)	(0.294)		
VSLA Support: Index: Baseline	2.594	2.633	0.051	1,041
	(1.002)	(1.109)		
**Main Outcomes**				
**Pursuit of Solar**				
Savings goal: Baseline	0.772	0.750	-0.023	1,041
	(0.420)	(0.433)		
Solar savings goal: Baseline	0.244	0.205	-0.042	1,041
	(0.430)	(0.404)		
Track savings goal: Baseline	0.276	0.280	-0.005	1,041
	(0.447)	(0.449)		
Expects to purchase this year: Baseline	0.728	0.655	-0.063	1,041
	(0.445)	(0.476)		
Panel or battery components: Baseline	0.061	0.048	-0.016	1,012
	(0.239)	(0.214)		
Lantern: no charging: Baseline	0.014	0.010	-0.004	1,012
	(0.118)	(0.098)		
Lantern: with charging: Baseline	0.061	0.039	-0.019	1,012
	(0.239)	(0.193)		
Solar home system: Baseline	0.105	0.091	-0.020	1,012
	(0.307)	(0.287)		
Don't know: Baseline	0.002	0.000	-0.002	1,012
	(0.045)	(0.000)		
Weekly solar savings: Total: Baseline	950.646	701.792	-277.346	1,012
	(3,106.311)	(2,668.387)		
**Solar Ownership**				
No solar device: Baseline	0.484	0.486	0.003	1,041
	(0.500)	(0.500)		
Panel or battery components: Baseline	0.264	0.250	-0.013	1,041
	(0.441)	(0.433)		
Lantern: no charging: Baseline	0.146	0.161	0.014	1,041
	(0.353)	(0.368)		
Lantern: with charging: Baseline	0.051	0.077	0.025	1,041
	(0.221)	(0.267)		

Solar home system: Baseline	0.051	0.023	-0.028*	1,041
	(0.221)	(0.148)		
Larger solar device: Baseline	0.102	0.098	-0.005	1,041
	(0.303)	(0.297)		

* This variable is a dummy constructed from a categorical variable (1 “not at all,” 2 “not really,” 3 “somewhat,” and 4 “very much”), combining 1 and 2 responses in the 0s and 3 and 4 responses in the 1s. At endline, the question is presented as a simple binary (0 “no” 1 “yes”). Thus, regressions on the aspirations outcome never control for the baseline levels of this variable due to the measurement change.
Notes: The table includes data from 1,041 baseline individuals collected during the baseline survey. Balance tests were conducted using t-tests, with Romano Wolf corrections for multiple hypothesis testing being run later (but not reported here). Column 2 reports the mean of control VSLAs and column 3 the mean of treatment VSLAs. Standard deviations are presented below in parentheses. Column 3 displays the difference between the two means, with standard errors displayed in parentheses. Asterisks denote a statistically significant difference at the 1% ***, 5% **, or 10% * levels.

Supplementary Analysis

Logit Regressions Corresponding to the OLS Analysis in the Paper

In this section on robustness checks, we first illustrate the robustness of our main OLS estimates (presented in Tables 5 and 6 in the main paper) to specifications for binary dependent variables. Supplementary Tables 5 and 6 below redo the OLS regressions presented in Tables 5 and 6 in the main paper using a logistic regression analysis. Marginal effect estimates are presented below.

Supplementary Table 5: ITT Pursuit of Solar Products (Logit)

	(1)<br>Savings goal	(2)<br>Solar savings goal	(3)<br>Tracks savings goal	(4)<br>Contacted company	(5)<br>Expects to purchase this year
Treatment VSLA	0.02	0.30***	0.02	0.07***	0.06
	(0.02)	(0.03)	(0.05)	(0.02)	(0.04)
Replacement	0.01	-0.13***	-0.02	-0.03	-0.00
	(0.02)	(0.05)	(0.05)	(0.03)	(0.05)
VSLA Controls	Yes	Yes	Yes	Yes	Yes
Individual Control	Yes	Yes	Yes	Yes	Yes
SEs Clustered VSLA	Yes	Yes	Yes	Yes	Yes
Observations	1186	1186	1186	1186	1186

Notes: Each column presents results of logit regressions for binary dependent variables on the binary treatment assignment variable. All regressions control for VSLA-level controls (a settlement dummy, a dummy for VSLAs that meet at least weekly, the number of members in the VSLA, and the VSLA's share price (normalized to weekly)) and individual-level controls (dummy for at least primary education). Robust standard errors clustered at the VSLA level are displayed below coefficients in parentheses. Asterisks denote a statistically significant difference at the 1% ***, 5% **, or 10% * levels.

Supplementary Table 6: ITT Ownership of Solar Devices (Logit)

	(1)<br>No solar device	(2)<br>Panel or battery components	(3)<br>Lantern: no charging	(4)<br>Lantern: with charging	(5)<br>Solar home system	(6)<br>Larger solar device	(7)<br>Acquired solar since intervention
Treatment VSLA	0.03	0.01	-0.01	-0.02	-0.01	-0.03	-0.03
	(0.04)	(0.03)	(0.03)	(0.01)	(0.02)	(0.03)	(0.02)
Replacement	-0.01	0.02	-0.02	0.02	-0.02	-0.00	-0.04
	(0.04)	(0.03)	(0.03)	(0.02)	(0.02)	(0.03)	(0.03)
VSLA Controls	Yes	Yes	Yes	Yes	Yes	Yes	Yes
Individual Control	Yes	Yes	Yes	Yes	Yes	Yes	Yes
SEs Clustered VSLA	Yes	Yes	Yes	Yes	Yes	Yes	Yes
Observations	1186	1186	1186	1186	1186	1186	1186

Notes: Each column presents results of logit regressions for binary dependent variables on the binary treatment assignment variable. All regressions control for VSLA-level controls (a settlement dummy, a dummy for VSLAs that meet at least weekly, the number of members in the VSLA, the VSLA's share price (normalized to weekly)), and individual-level controls (dummy for at least primary education). Robust standard errors clustered at the VSLA level are displayed below coefficients in parentheses. Asterisks denote a statistically significant difference at the 1% ***, 5% **, or 10% * levels.

ANCOVA Analysis: Controlling for Baseline Levels of Outcome Variables

Next, we present regressions run using our ANCOVA specification as described in the empirical section, in which the baseline value of the outcome variable is controlled for in each regression. Note that, as described, values of the baseline level of the outcome variables are set to zero for those individuals for whom we do not have baseline surveys, and a dummy variable identifying those without baselines is then also controlled for.

This specification is not our preferred for the study, given the need to control for the missing baseline surveys in order to estimate outcomes. Since the one-wave OLS specification presented in the main body of the paper does not rely on baseline data at all, there is less chance of those estimates being biased due to this missing data. Furthermore, we do not have two waves of data for any respondents for some of our outcomes of interest, and thus could not run two wave regressions for impacts on the amount saved weekly with VSLA, the amount saved weekly outside of the VSLA, contacting solar companies, or having acquired a new solar device. For this reason, ANCOVA and DID regressions are presented here in the supplementary materials.

Results of the ANCOVA regressions match those found in the OLS regressions in the main paper. See Supplementary Tables 7-9 below.

Supplementary Table 7: ITT Pursuit of Solar Products (ANCOVA)

	(1)<br>Savings goal	(2)<br>Solar savings goal	(3)<br>Tracks savings goal	(4)<br>Expects to purchase this year
Treatment VSLA	0.02	0.33***	0.02	0.07*
	(0.02)	(0.04)	(0.04)	(0.04)
Replacement	0.02	-0.11**	0.07	0.07
	(0.03)	(0.04)	(0.05)	(0.06)
Savings goal: Baseline	0.01			
	(0.02)			
Solar savings goal: Baseline		0.08*		
		(0.04)		
Tracks savings goal: Baseline			0.29***	
			(0.04)	
Expects to purchase this year: Baseline				0.12***
				(0.04)
Constant	0.83***	0.21***	0.45***	0.51***
	(0.05)	(0.07)	(0.08)	(0.08)
VSLA Controls	Yes	Yes	Yes	Yes
Individual Controls	Yes	Yes	Yes	Yes
SEs Clustered VSLA	Yes	Yes	Yes	Yes
R-squared	0.03	0.21	0.08	0.02
Observations	1186	1186	1186	1186

Notes: Each column presents results of OLS regressions for binary dependent variables on the binary treatment assignment variable, controlling for the baseline level of the dependent variable. Results are ITT estimates for those respondents for whom we have baseline data due to imperfect intervention compliance and incomplete baseline data collection. All regressions control for VSLA-level controls (a settlement dummy, a dummy for VSLAs that meet at least weekly, the number of members in the VSLA, and the VSLA's share price (normalized to weekly)), and individual-level controls (dummy for at least primary education). Robust standard errors clustered at the VSLA level are displayed below coefficients in parentheses. Asterisks denote a statistically significant difference at the 1% ***, 5% **, or 10% * levels.

Supplementary Table 8: ITT Ownership of Solar Devices (ANCOVA)

	(1) No solar device	(2) Panel or battery components	(3) Lantern: no charging	(4) Lantern: with charging	(5) Solar home system	(6) Larger solar device
Treatment VSLA	0.04	0.01	-0.01	-0.02	-0.00	-0.03
	(0.04)	(0.02)	(0.03)	(0.01)	(0.01)	(0.03)
Replacement	0.14***	0.08**	-0.00	0.02	-0.00	0.01
	(0.05)	(0.03)	(0.03)	(0.02)	(0.02)	(0.04)
No solar device: Baseline	0.30***					
	(0.03)					
Panel or battery components: Baseline		0.23***				
		(0.04)				
Lantern: no charging: Baseline			0.08**			
			(0.04)			
Lantern: with charging: Baseline				0.05		
				(0.04)		
Solar home system: Baseline					0.29***	
					(0.11)	
Larger solar device: Baseline						0.07
						(0.05)
Constant	0.48***	-0.00	0.16***	0.08***	0.06***	0.26***
	(0.06)	(0.04)	(0.05)	(0.03)	(0.02)	(0.05)
VSLA Controls	Yes	Yes	Yes	Yes	Yes	Yes
Individual Controls	Yes	Yes	Yes	Yes	Yes	Yes
SEs Clustered VSLA	Yes	Yes	Yes	Yes	Yes	Yes
R-squared	0.10	0.07	0.01	0.02	0.06	0.03
Observations	1186	1186	1186	1186	1186	1186

Notes: Each column presents results of OLS regressions for binary dependent variables on the binary treatment assignment variable, controlling for the baseline level of the dependent variable. Results are ITT estimates for those respondents for whom we have baseline data due to imperfect intervention compliance and incomplete baseline data collection. All regressions control for VSLA-level controls (a settlement dummy, a dummy for VSLAs that meet at least weekly, the number of members in the VSLA, the VSLA's share price (normalized to weekly)), and individual-level controls (dummy for at least primary education). Robust standard errors clustered at the VSLA level are displayed below coefficients in parentheses. Asterisks denote a statistically significant difference at the 1% ***, 5% **, or 10% * levels.

Supplementary Table 9: ITT Pursuit of Solar Products (Tobit - ANCOVA)

	(1)<br>Panel or battery components	(2)<br>Lantern: with charging	(3)<br>Solar home system	(4)<br>Weekly solar savings: Total
Treatment VSLA	0.04**	0.09***	0.13***	2,112.61***
	(0.02)	(0.03)	(0.03)	(289.67)
Panel or battery components: Baseline	0.05*			
	(0.03)			
Replacement	-0.01	-0.07*	-0.05	-755.23**
	(0.02)	(0.04)	(0.03)	(377.78)
Lantern: with charging: Baseline		0.15***		
		(0.03)		
Solar home system: Baseline			0.09***	
			(0.04)	
Weekly solar savings: Total: Baseline				0.13***
				(0.04)
VSLA Controls	Yes	Yes	Yes	Yes
Individual Controls	Yes	Yes	Yes	Yes
SEs Clustered VSLA	Yes	Yes	Yes	Yes
Observations	1159	1159	1159	1157

Notes: Each column presents results of Tobit regressions for dependent variables on the binary treatment assignment variable, controlling for the baseline level of the dependent variable. Dependent variables in columns 1-3 are binary, and in column 4 are continuous. Results are ITT estimates for those respondents for whom we have baseline data due to imperfect intervention compliance and incomplete baseline data collection. All regressions control for VSLA-level controls (a settlement dummy, a dummy for VSLAs that meet at least weekly, the number of members in the VSLA, the VSLA's share price (normalized to weekly)), and individual-level controls (dummy for at least primary education). Robust standard errors clustered at the VSLA level are displayed below coefficients in parentheses. Asterisks denote a statistically significant difference at the 1% ***, 5% **, or 10% * levels.

Difference in Differences Analysis

We present the regressions run using our DID specification, as described in the empirical section below.

This specification is not our preferred one for the study, as the missing baseline data mentioned above likely leads to a selected sample. However, the results of the DID regressions match those found in the OLS and the ANCOVA regressions. In addition, coefficients for expecting to purchase a solar device (+) and for owning a lantern with charging facilities (-) are significant in this specification only (rather than close to significant, as in the others).

Supplementary Table 10: ITT Pursuit of Solar Products (DID)

	(1) Savings goal	(2) Solar savings goal	(3) Tracks savings goal	(4) Expects to purchase this year
Treatment VSLA*Endline	0.04	0.36***	0.02	0.13**
	(0.05)	(0.05)	(0.05)	(0.06)
Treatment VSLA	-0.02	-0.04	0.00	-0.07*
	(0.04)	(0.04)	(0.04)	(0.04)
Endline	0.14***	0.04	0.25***	-0.10**
	(0.03)	(0.04)	(0.04)	(0.04)
Constant	0.77***	0.24***	0.28***	0.73***
	(0.03)	(0.03)	(0.03)	(0.03)
VSLA Controls	No	No	No	No
Individual Controls	No	No	No	No
SEs Clustered VSLA	Yes	Yes	Yes	Yes
Observations	2243	2243	2243	2243

Notes: Each column presents results of DID regressions for binary dependent variables on the binary treatment assignment variable, a dummy for the endline, and the interaction between the two. Results are ITT estimates for those respondents for whom we have baseline data due to imperfect intervention compliance and incomplete baseline data collection. Robust standard errors clustered at the VSLA level are displayed below coefficients in parentheses. Asterisks denote a statistically significant difference at the 1% ***, 5% **, or 10% * levels.

Supplementary Table 11: ITT Ownership of Solar Devices (DID)

	(1) No solar device	(2) Panel or battery components	(3) Lantern: no charging	(4) Lantern: with charging	(5) Solar home system	(6) Larger solar device
Treatment VSLA*Endline	0.05	0.02	-0.03	-0.05**	0.02	-0.04
	(0.04)	(0.03)	(0.03)	(0.02)	(0.02)	(0.04)
Treatment VSLA	-0.00	-0.02	0.02	0.02	-0.03*	0.00
	(0.04)	(0.03)	(0.03)	(0.02)	(0.01)	(0.02)
Endline	0.06**	-0.12***	0.03	0.02	0.00	0.15***
	(0.03)	(0.02)	(0.03)	(0.01)	(0.01)	(0.03)
Constant	0.48***	0.27***	0.14***	0.05***	0.05***	0.10***
	(0.03)	(0.02)	(0.02)	(0.01)	(0.01)	(0.02)
VSLA Controls	No	No	No	No	No	No
Individual Controls	No	No	No	No	No	No
SEs Clustered VSLA	Yes	Yes	Yes	Yes	Yes	Yes
Observations	2243	2243	2243	2243	2243	2243

Notes: Each column presents results of DID regressions for binary dependent variables on the binary treatment assignment variable, an endline dummy, and the interaction between the two. Results are ITT estimates for those respondents for whom we have baseline data due to imperfect intervention compliance and incomplete baseline data collection. Robust standard errors clustered at the VSLA level are below coefficients in parentheses. Asterisks denote statistically significant differences at the 1% ***, 5% **, or 10% * levels.

Supplementary Table 12: ITT Pursuit of Solar Products (Tobit - DID)

	(1) Panel or battery components	(2) Lantern: with charging	(3) Solar home system	(4) Weekly solar savings: Total
Treatment VSLA*Endline	0.04**	0.09***	0.13***	1,894.64***
	(0.02)	(0.03)	(0.03)	(267.59)
Treatment VSLA	-0.01	-0.03	-0.02	-327.53
	(0.01)	(0.02)	(0.02)	(213.21)
Endline	-0.02	0.06***	-0.03*	614.45***
	(0.01)	(0.02)	(0.02)	(190.68)
VSLA Controls	No	No	No	No
Individual Controls	No	No	No	No
SEs Clustered VSLA	Yes	Yes	Yes	Yes
Observations	2214	2214	2214	2212

Notes: Each column presents results of panel Tobit regressions to estimate the impact on dependent variables of the binary treatment assignment variable, a dummy for the endline, and the interaction between the two. Dependent variables in columns 1-3 are binary, and in column 4 are continuous. Results are ITT estimates for those respondents for whom we have baseline data due to imperfect intervention compliance and incomplete baseline data collection. Robust standard errors clustered at the VSLA level are below coefficients in parentheses. Asterisks denote a statistically significant difference at the 1% ***, 5% **, or 10% * levels.

Multiple Hypothesis Testing

Supplementary Table 13 presents the Romano Wolf adjusted p-values for the regressions presented in this report. OLS and Tobit specifications are used in accordance with reporting in the main body of the document. The significance of the estimated outcomes does not change when accounting for the multiple hypotheses tested.

Supplementary Table 13. Romano Wolf P-Values: Adjusted for Multiple Hypothesis Testing

**Variables**	**Original P-Value**	**Resample P-Value**	**Romano Wolf P-Value**	**Significance Level**
**Intermediate Outcomes**				
Knowledge of Targeted Solar Benefits: Index	0	.0099	.0099	***
Knowledge of Certified Products: Index	0	.0099	.0099	***
Trust in Solar Providers: Index	.0023	.0099	.0099	***
Aspire to purchase solar this year	.0271	.0099	.0099	***
Self-Efficacy: Index	.2292	.0594	.3762	
VSLA Support: Index	0	.0099	.0099	***
**Outcomes**				
**Pursuit of Solar**				
Savings goal	.2646	.0693	.4158	
Solar savings goal	0	.0099	.0099	***
Track savings goal	.6254	.3861	.802	
Contacted company	.0029	.0099	.0099	***
Expects to purchase this year	.1219	.0099	.1782	
Panel or battery components	.0537	.0099	.0099	***
Lantern: no charging	0	.0099	.0099	***
Lantern: with charging	.001	.0099	.0099	***
Solar home system	0	.0099	.0099	***
Weekly solar savings: VSLA	0	.0099	.0099	***
Weekly solar savings: Other	.0003	.0099	.0099	***
Weekly solar savings: Total	0	.0099	.0099	***
**Solar Ownership**				
No solar device	.4086	.1287	.5545	
Panel or battery components	.6539	.4653	.802	
Lantern: no charging	.6568	.4752	.802	
Lantern: with charging	.1956	.1089	.3564	
Solar home system	.5748	.3465	.802	
Larger solar device	.2711	.099	.4158	
Acquired solar since intervention	.261	.0792	.4158	

Notes: Corrections for multiple hypothesis testing are carried out using Romano Wolf adjusted p-values. Asterisks denote a statistically significant difference at the 1% ***, 5% **, or 10% * levels.

Supplementary Discussion (1)

Linking Intermediate Outcomes, Solar Savings, and Solar Purchases

To consider the likelihood that the outcomes we observe in the short term will lead to purchases of solar products in the longer term, we consult related literature on links between intentions, initial (self-reported) behaviors (saving, in our case), and final behaviors (purchases).

Intentions

A significant amount of work has looked at links between variables similar to our intermediate outcomes – such as knowledge, attitudes, and self-efficacy – and the adoption of energy-efficient technologies. Most of this literature bases models of purchase intentions on variants of the theory of planned behavior.[1] Considerable evidence suggests that the components of the theory do lead to purchase intentions for new technologies. However, the majority of these studies rely on purchase intentions as their outcome without measuring final incidences of device purchase. Literature on the links between these intermediate variables and the final outcome is scarce.

When it comes to links between purchase intentions and actions, the literature on willingness to pay offers some insights by comparing stated and revealed preferences for new technologies, using willingness-to-pay measures and real purchases. This work tends to find differences between participant willingness to pay and actual purchase behavior, attributing the gap to a combination of overconfidence, liquidity constraints, and weak enforcement.[2,3,4]

However, our study measures respondents' self-reported savings, which is a behavior in pursuit of solar rather than merely an expression of purchase intentions or willingness to pay. Assuming respondents are answering as truthfully as they are able to (we relax this assumption to consider the possibility of social desirability bias in the conclusion and in Supplementary Discussion 2), we consider to what extent (i) savings self-reports are likely to be accurate and (ii) how confident we can be that these will result in solar purchases.

Accuracy of Reports

Literature on the accuracy of savings self-report measures in the developing world is scarce, with existing work tending to focus on wealthier nations and individuals with access to formal financial mechanisms. This work suggests some discrepancies between the stock of savings individuals report relative to those they actually have. However, this finding comes primarily from the extensive margin – a lack of reporting of certain accounts – with reports about funds within specific accounts being accurate.[5] Since our work focuses on specific savings mechanisms, we are less concerned about this type of inaccuracy. Work on savings goal realism in Canada finds that larger goals can lead to an underestimation of future expenditure by savers.[6] Such a discrepancy would likely cause difficulties complying with savings goals when faced with the reality of future expenses. However, the study in question was carried out among students who still depended on their parents for their income and used it only for discretionary spending. For this reason, it is not a comparable context to refugees living in extreme poverty, who likely pay considerably more attention to their available income.

Work in developing countries acknowledges the potential challenges posed by the need to rely on self-reported data in the context of informal savings mechanisms and notes that small flows

of savings can be less salient than larger flows, and may therefore be more difficult to accurately remember.[7] However, no concrete evidence exists to say that systematic biases exist between self-reports and more observational measures in this context.

Indeed, research on financial management by the poor, and particularly on the effects of scarcity, suggests that scarcity can lead to greater attention being paid to financial decisions and thus to more consistent choices.[8] Conversely, there is also evidence to suggest scarcity can increase focus on the present, with lesser concern for the future impact of decisions. Due to a significant lack of evidence (and especially evidence from the field), it is hard to judge what the exact relationship between self-reported savings, actual savings, and future savings is likely to be in this context.

Likelihood of Actual Purchases

The literature is also lacking when it comes to links between savings goals and goal achievement, as it tends to focus on outcomes such as savings amounts or expected achievement rather than goal actualization. However, in a study measuring impacts of public commitment within savings groups on savings goal achievement, Salas (2022) finds that those who publicly committed to savings goals were 8.5% more likely to achieve them.[9] Given the similarities between the design of this study and our own, we are hopeful that the act of setting and committing to goals within an VSLA will help savers in our study achieve their goals when possible.

The studies that investigate impacts on the perceived ease of achieving goals, rather than actual goal achievement, argue that savings goals for a particular purpose are expected to be more successful if they are specific (as in the amount to be put aside), as was the case in our study.[10] Note that, though it can affect savings intentions and amounts, the number of goals doesn't seem to impact perceived likelihood of goal achievement.[11]

Our Sample

In our sample, due to the short reporting periods and focus on one savings mechanism, we are inclined to expect self-reported VSLA savings to be accurate. However, the likelihood of a conversion from savings to purchases in our sample is hard to glean from existing studies and will depend both on the internal variables and external factors specific to the context in which we are working. Though we are hopeful that the characteristics of the savings goals made will aid goal achievement (realistic, specific, publicly committed to), it is possible that—as we observe for some respondents between intervention and endline—some refugees may need goal flexibility over time or, in more extreme circumstances, will have to set their goals aside.

Given the obstacles to saving experienced by the poor and marginalized,[12] we judge the flexibility offered by VSLAs to be key for refugees to safely explore the feasibility of solar savings goals. Especially when compared to the high interest rates and inflexible payment schedules of PAYG solar financing, VSLA savings offer a low-risk method of working toward solar products at a sustainable pace. Given this, we expect those who reach their solar goal to do so without large sacrifices and for those with more immediate priorities to be able to address them without painful social or financial consequences.

Supplementary Discussion (2)

Savings Goals Specifics

This section examines the savings goals set by treatment participants in more detail, specifically considering the possibility of over-commitment and social desirability bias. Data collected during the intervention itself is used for this exercise, alongside reports about savings goals at endline.

Savings Goal Type

Among the intervention attendees, 521 of 537 reported setting saving goals during the intervention (97%). Of these goals, 77% (399) were for solar products, with the rest (23%) being for non-solar goals, such as the payment of school fees, medical expenses, or livestock purchases. The prevalence of non-solar goals assuages some concerns about social desirability bias, since it seems to have been clear that savings goals for any purpose were welcome at the session.

Respondents with solar goals most commonly reported saving for solar lanterns (including both light and charging capabilities) (34.34%) and solar home systems (30.58%), followed by solar lanterns (only lights) (19.8%).

Time To Reach Savings Goals

The time respondents expect to take to achieve their savings goals is reported in Supplementary Figure 8 below. This variable helps us start to gauge the realism of respondents' solar savings goals.

On average, respondents expect to be saving for 12-13 more months to achieve their goals. This appreciation of the fact that solar goals will take significant time to achieve suggests a degree of realism. Note, the fact that all three distributions have local maxima around 12 months, 24 months, and 36 months could indicate either rounding bias or reflect the length of VSLA savings cycles (~12 months).

Further, the time respondents expect to take to achieve their goal varies by the type of solar product they are aiming to purchase. Supplementary Figure 9 below displays these expectations by solar product type. Savings goals for simple solar lanterns (with only a light) are expected to take an additional 6-7 months to be fulfilled, on average, while more advanced solar lanterns (with plugs for charging) and solar home systems are expected to take a median of 12 additional months to achieve. Moreover, while these two categories have similar median values, it is important to note the right-skewed nature in the distribution for advanced solar lanterns in contrast to the left-skew of the median for solar home systems. The higher inter-quartile range of saving times for those aiming to purchase solar home systems is consistent with the availability of a wide range of such systems at varying price points.

Supplementary Figure 8: Savings Goal Expected Months Remaining

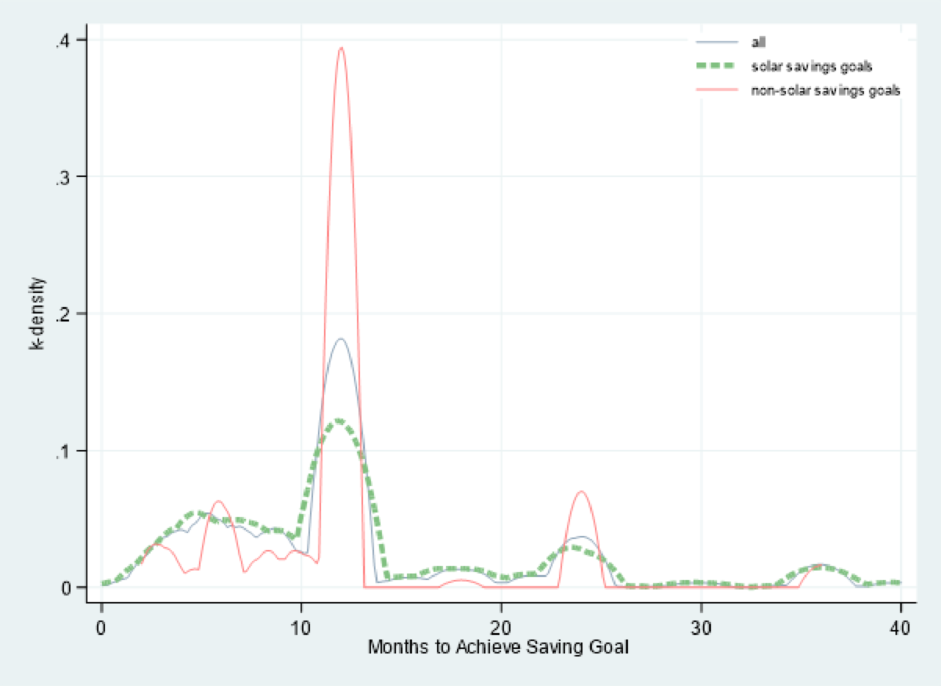

Notes: Supplementary Figure 8 presents the distribution of the expected months remaining for respondents' savings goals to be achieved. Data collected at endline was used to generate the figure.

Supplementary Figure 9: Average Expected Savings Months Remaining

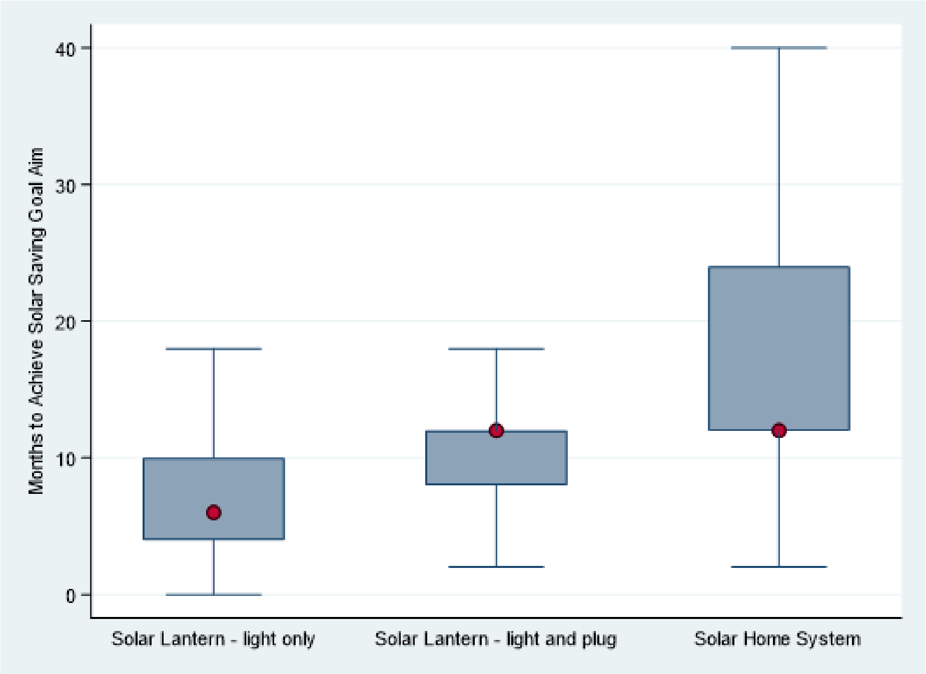

Notes: Supplementary Figure 9 presents the distribution of the expected months remaining for respondents' solar savings goals to be achieved, separated by solar product type. Data collected at endline was used to generate the figure.

Changes to Solar Goals Between Intervention and Endline

It appears that respondents were willing to adjust their savings goals to reflect their financial situation over time. Indeed, when we inquired about solar savings goals at endline, 291 of the respondents reported still having such a goal (73% of those who set one initially). This reduction was uniformly distributed across all reported solar product savings goal categories during treatment. Furthermore, at least 30% of respondents adjusted their solar savings goals upwards or downwards over time. Reports of goal abandonment and adjustment increase our confidence in the reliability of the measures of solar savings, since they demonstrate that respondents feel able to report behavior they may not necessarily consider to be "socially desirable." They also suggest that participants do not necessarily feel bound to their goals after informal commitment, assuaging some concern about over-commitment.

Supplementary Table 14: Changes to Solar Goals Between Intervention and Endline

Solar Product as Saving Goal	Reported During Treatment Rollout	Change at Endline			Unchanged
		Total	Upgrade	Downgrade	
Solar Lantern – light only	51	14 (27%)	14 (27%)	-	34 (67%)
Solar Lantern – light and plug	103	39 (38%)	12 (12%)	27 (26%)	55 (53%)
Solar Home Systems	91	23 (25%)	-	23 (25%)	60 (66%)
Total	245	76 (31%)	26 (11%)	50 (20%)	149 (61%)

Notes: Supplementary Table 14 presents the precise statistics for goal adjustments between the intervention and the endline. It only includes respondents who report saving for a solar lantern (simple or with charging) or a solar home system, since the solar panel or battery component (n=46) is not possible to rank in terms of size/cost. As a result, any shortfall in percentage totals in each category can be attributed to changes in the omitted category.

Comparing Measures of Solar Goals

Finally, we consider the consistency between respondents' stated solar goals and their reported savings behavior, as another test of goal realism (as well as perhaps a way of evaluating social desirability). This exercise involves comparing the remaining savings needed to achieve respondents' solar goals (how much more they need to save to achieve this goal) to their planned future saving for this goal (the amount they are putting aside each week multiplied by the amount of time they plan to do so).

Supplementary Figure 10 presents the distribution of the differences between these two variables, showing that the values were near-identical to each other in most cases (the median difference is 1,000 UGX, or $0.26). The comparability of these two distributions assuages our concerns about overconfidence/unrealistic goal setting, since respondents have clearly calculated effectively what their savings goals will entail.

To conclude, we consider the number of non-solar goals, the relatively common abandonment or adjustment of solar goals to better suit refugees' needs, and the accuracy of expectations about goal timings to be indicative of thought, realism, and adaptability on the part of savers. These traits increase our faith in the reliability of the responses collected and thus lessen concerns about social desirability bias and overconfidence.

Supplementary Figure 10: Difference Between Amount Left to Save Measured in Two Ways

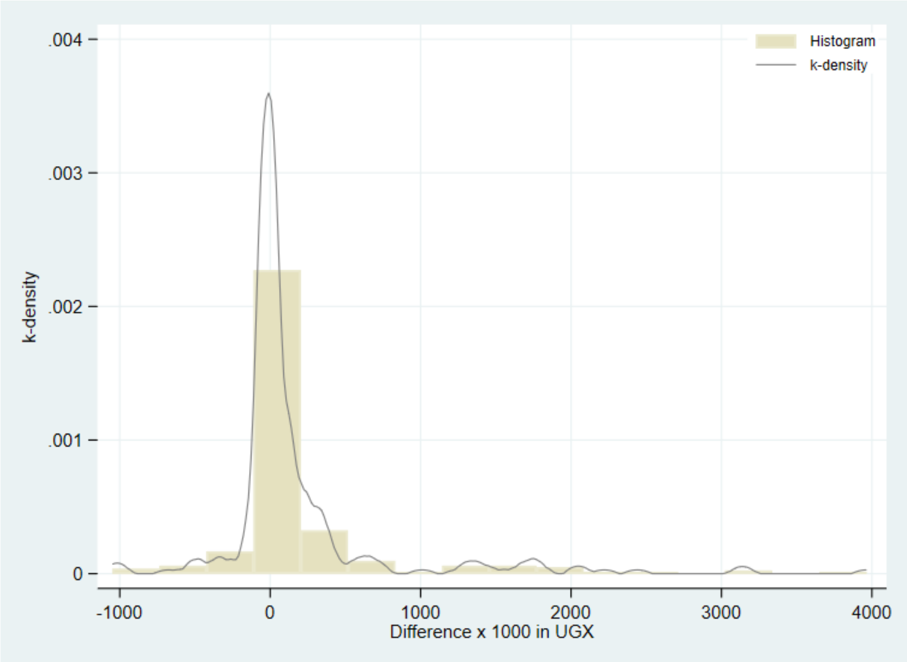

Notes: Supplementary Figure 10 presents the distribution of the differences between two measures of the amount respondents have left to save to achieve their solar savings goal. Data collected at endline was used to generate the figure.

Supplementary Discussion (3)

To determine the possible implications of increased solar saving for refugees, we discuss the financial context in which this occurred for households. In particular, we ask whether increased solar saving occurred as a complement to or a substitute for other forms of saving in our sample. We cannot measure the precise tradeoffs for savers, but an analysis of total savings amounts and of other reported savings goals helps us to think through the context of choices made by households.

Supplementary Table 15 summarizes this analysis, focusing on the impacts on total savings, the number of savings goals reported, and the existence of various other savings goals. We cannot reject the null hypothesis of no change to total saving or to the number of savings goals individuals were working toward in the treatment group. Thus, it seems that increases in solar savings at least partly represent decreases in other types of saving. Though we are unable to calculate the optimal savings portfolio for each refugee based on their resources or the amount they already have saved for other goals, we can see which goals those in treatment VSLAs are more or less likely to be pursuing at the moment the endline survey was collected. The results show a reduced likelihood that these individuals report specific savings goals for emergencies, non-solar assets, non-solar businesses, or school fees.

We do not have sufficient data to determine whether the lack of an increase in total savings alongside solar savings in treatment VSLAs is a sign of an optimal shift or one that could present challenges for savers down the line. Likewise, an unchanged number of savings goals in the context of higher saving for solar could be a positive, neutral, or negative sign, depending on the link between savings need, number of goals, amount of total saving and any trade-offs between savings for different purposes. For instance, this could reflect a positive shift in the short-term to prioritize solar savings that leads refugees to meet this goal more quickly than if they were saving for many goals at once[11]. However, it could also indicate a shift away from savings “pots,” which could present challenges when it comes dealing with other important (expected or unexpected) expenses.

Though it is not possible to know the full implications of increased solar saving among intervention participants, the study’s selection criteria may have guarded against encouraging solar saving from those unlikely to benefit. Indeed, study participants were only eligible to participate if they were already interested in saving towards a solar device that was “larger than a simple lantern (For example: i) a lantern that allows for phone charging, ii) a system that allows for more than one light and charging or, iii) a system that powers lights and an appliance like a television or a radio).” We expect that individuals who answered “yes” to this screening question were more likely than individuals who answered “no” to consider themselves able to set aside savings for solar without negative consequences. However, it is still conceivable that an individual for whom saving for solar would be challenging or have negative consequences could have provided a positive response to this question.

While we cannot ascertain the full impact of increased saving for solar in this context, we did ask participants whether they faced challenges in sticking to their savings goals in the face of unexpected expenses. There is no evidence to suggest that those from treatment VSLAs experienced increased difficulty in doing so (we cannot reject the null hypothesis of no impacts of treatment on this variable – regression (13)). This could indicate that treated savers are equally likely to consider themselves able to use their savings for emergencies since setting a solar savings goal. However, it does not allow us to measure impacts on other types of expected expenditures.

Related to the question of unexpected expenditure, our Supplementary Discussion (2) considers refugee willingness to adapt savings goals to changing circumstances. This descriptive analysis helps us evaluate the extent to which savings goals were flexible when necessary, an important characteristic of saving in the context of unpredictable income streams. We find that some savers do adjust their goals over time (both upward and downward). This reassures us that refugees would be able to resort to this option if needed.

Future studies on this topic could try to causally determine whether increased saving for solar represents a more efficient allocation of resources or a sacrifice that could create challenges down the line. Alternatively, to guard against any crowding out that might have negative effects, future work could support participants in planning their savings portfolio generally rather than focusing on one element of savings, or test the effect of solar savings information in the context of broader savings interventions (where other goals are given equal weight). Additionally, it would be interesting to evaluate interventions that combine solar savings content with some form of financial support to refugees to see whether this results in an increase in total savings in addition to savings toward solar goals. These approaches would help to evaluate whether increases in solar savings are optimal for participants, given their other priorities.

Supplementary Table 15: Broader Savings Context

	(1) Average weekly savings	(2) Number of savings goals	(3) Goal: none	(4) Goal: emergencies	(5) Goal: daily spendin g	(6) Goal: solar	(7) Goal: non-solar asset	(8) Goal: non-solar business	(9) Goal: return home	(10) Goal: un-specified	(11) Goal: school fees	(12) Goal: other	(13) Goals hard when un-expected expenses
Treatment VSLA	5.06	-0.03	-0.01	-0.09**	0.01	0.32***	-0.05**	-0.06***	-0.01	-0.02	-0.12***	-0.02	-0.06
	(557.52)	(0.08)	(0.01)	(0.04)	(0.04)	(0.04)	(0.02)	(0.02)	(0.01)	(0.02)	(0.03)	(0.02)	(0.04)
Replacement	-402.49	-0.05	0.00	0.04	-0.03	-0.13***	-0.01	0.02	-0.00	0.01	0.07	-0.03	0.05
	(647.01)	(0.09)	(0.01)	(0.04)	(0.05)	(0.04)	(0.02)	(0.03)	(0.01)	(0.03)	(0.05)	(0.02)	(0.05)
Constant	5,128.94***	1.34***	0.02	0.12*	0.24***	0.23***	0.07**	0.19***	0.02	0.19***	0.16***	0.12**	0.25***
	(1,059.81)	(0.12)	(0.01)	(0.06)	(0.07)	(0.07)	(0.03)	(0.05)	(0.02)	(0.05)	(0.05)	(0.06)	(0.09)
VSLA Controls	Yes	Yes	Yes	Yes	Yes	Yes	Yes	Yes	Yes	Yes	Yes	Yes	Yes
Individual Control	Yes	Yes	Yes	Yes	Yes	Yes	Yes	Yes	Yes	Yes	Yes	Yes	Yes
SEs Clustered VSLA	Yes	Yes	Yes	Yes	Yes	Yes	Yes	Yes	Yes	Yes	Yes	Yes	Yes
R-squared	0.01	0.23	0.00	0.21	0.03	0.21	0.02	0.09	0.01	0.03	0.26	0.03	0.05
Observations	1174	1186	1186	1186	1186	1186	1186	1186	1186	1186	1186	1186	1186

Notes: Each column presents results of OLS regressions for dependent variables on the binary treatment assignment variable. The outcomes in columns (1) and (2) are continuous, and the rest of the outcomes are binary. Results are ITT estimates due to imperfect intervention compliance. All regressions control for VSLA-level controls (a settlement dummy, a dummy for VSLAs that meet at least weekly, the number of members in the VSLA, and the VSLA's share price (normalized to weekly)), and individual-level controls (dummy for at least primary education). Robust standard errors clustered at the VSLA level are displayed below coefficients in parentheses. Asterisks denote a statistically significant difference at the 1% ***, 5% **, or 10% * levels.

Supplementary References

1. Fathima, A., Batcha, M. and Alam, A. Factors affecting consumer purchase intention for buying solar energy products. *International Journal of Energy Sector Management* **17(4)**, 820-839, (2023)
2. Grimm, M., Lenz, L., Peters, J. and Sievert, M. Demand for off-grid solar electricity: Experimental evidence from Rwanda. *Journal of the Association of Environmental and Resource Economists* **7(3)**, 417-454, (2020).
3. Reynolds, T., Murray, B., Kolodinsky, J. and Howell, J. Contrasting self-reported willingness to pay and demonstrated purchase behavior for energy-saving technologies in a small island developing state. *Energy for Sustainable Development* **27,** 18-27, (2015).
4. Mobarak, A., Dwivedi, P., Bailis, R., Hildemann, L. and Miller, G. Low demand for nontraditional cookstove technologies. *Proceedings of the National Academy of Sciences* **109(27)**, 10815-10820, (2012).
5. Ferber, R., Forsythe, J., Guthrie, H. and Maynes, E.S. Validation of a national survey of consumer financial characteristics: savings accounts. *The Review of Economics and Statistics, **51(4)**,* 436-444, (1969).
6. Peetz, J., and Buehler, R. Is there a budget fallacy? The role of savings goals in the prediction of personal spending. *Personality and Social Psychology Bulletin,* **35(12)**, 1579-1591, (2009).
7. Karlan, D., Ratan, A.L. and Zinman, J. Savings by and for the Poor: A Research Review and Agenda. *Review of Income and Wealth* **60(1)**, 36-78, (2014).
8. De Bruijn, E.J., and Antonides, G. Poverty and economic decision making: a review of scarcity theory. *Theory and Decision* **92(1)**, 5-37, (2022).
9. Salas Bahamón, L.M. Inclusión financiera en Colombia. Evaluación de impacto del programa Grupos de Ahorro y Crédito Comunitario. *Cuadernos de Economía*, **41(87)**, 747-782, (2022).
10. Ülkümen, G, and Cheema, A. Framing goals to influence personal savings: The role of specificity and construal level. *Journal of marketing research* **48(6)**, 958-969, (2011).
11. Soman, D., and Zhao, M. The fewer the better: Number of goals and savings behavior. *Journal of Marketing Research* **48(6)**, 944-957, (2011).
12. Banerjee, A.V., and Duflo, E. The economic lives of the poor. *Journal of economic perspectives* **21(1)**, 141-167, (2007).
